# A pilot randomized clinical trial of biomedical link with mental health in art therapy intervention programs for alcohol use disorder: Changes in NK cells, addiction biomarkers, electroencephalography, and MMPI-2 profiles

**DOI:** 10.1371/journal.pone.0284344

**Published:** 2023-05-05

**Authors:** Soo-Ji Kang, Chang-Zhu Pei, Da-Hye Lee, Jong-Eun Ha, Kwang-Hyun Baek

**Affiliations:** 1 Department of Medicine in General Graduate School, CHA University, Gyeonggi-Do, Republic of Korea; 2 Department of Biomedical Science, CHA University, Gyeonggi-Do, Republic of Korea; 3 KARF St. Mary’s Hospital, Gyeonggi-Do, Republic of Korea; University of Cincinnati Clermont College, UNITED STATES

## Abstract

**Objective:**

Alcohol intake is a major risk factor for various diseases. Elucidating alcohol use disorder (AUD) is important in preventing diseases and promoting health. We aimed to investigate the effect of art therapy on emotional (Minnesota Multiphasic Personality Inventory-2 [MMPI-2]) and physical (natural killer [NK] cell count, expression of stress-associated proteins [SAP], and electroencephalography) changes in patients with AUD.

**Methods:**

Participants were randomly divided into two groups (n = 35), with the experimental group undergoing art therapy involving weekly 60-min group therapy sessions for 10 weeks. Statistical analysis was performed using Ranked ANCOVA and Wilcoxon’s signed rank test. Western blotting was performed to analyze serum SAP levels.

**Results:**

We observed an association between psychological mechanisms and stress proteins. There was an increased number of NK cells in the experimental group after the program. Moreover, compared with the control group, the experimental group showed significant changes in SAP expression. Further, the experimental group showed a positive change in the MMPI-2 profile, as well as a decrease in depression, anxiety, impulsivity, and alcohol dependence.

**Conclusions:**

Continuous psychological support could be applied as a stress-control program for preventing stress recurrence and post-discharge relapse. Our findings strengthen the link between biomedical science and mental health in rehabilitation treatment for AUD.

## Introduction

According to the World Health Organization, alcohol is the most consumed psychoactive substance worldwide, with an estimated 3 million deaths annually worldwide due to harmful alcohol use [1,2]. Alcohol use disorder (AUD) refers to a maladaptive disorder caused by excessive alcohol use. It is a chronic and progressive psychiatric disorder that causes loss of control and results in physical, psychological, social, and legal problems [3].

Chronic alcohol use causes extensive structural, functional, and neurobiological changes in the nervous system, leading to a various problems including addiction, thinking, emotional, cognitive and behavioral disorders [4]. Therefore, there is a need for valid and useful research on the link between biomedical and mental health. Art therapy (AT), which is a form of complementary and alternative medicine, has been used to treat addiction since the 1950s [5]. AT allows patients to express their inner world through creative and diverse art activities and enables behavioral activation during the AT process. It is also a therapy for positive change emotionally, physically, spiritually, and socially. AT combined with other recovery services, including addiction detoxification, individual therapy, group therapy, and family counseling, can accelerate the healing process.

### AUD, stress proteins, immune cells, and electroencephalography (EEG) changes

Prolonged alcohol use significantly decreases leukocyte levels and antibody production. In case of long-term alcohol misuse, alcohol directly acts on tissues involved in stress response, which increases the production of stress-related proteins [6]. In addition, patients with AUD present with significantly inhibited immune functions, which increases their susceptibility to bacterial or viral infections [6]. Biomarkers for AUD can identify the current disease state and provide useful prognostic information [7].

In addition to biomarkers, changes in EEG are also important for AUD patients. Brain injury caused by chronic alcohol consumption involves deteriorated brain signaling, physiological and psychological tension through stimulation of the autonomic nervous system, and withdrawal symptoms such as anxiety and sleep disorders [8–10]. Since the fundamental problem of alcohol-related psychiatric symptoms involves brain dysfunction, neurophysiological studies using the brain waves of patients with AUD can provide noninvasive and stable information [11]. Further, technological advances have allowed accurate quantitative electroencephalography; accordingly, studies can assess cerebral functions or utilize neurofeedback to examine patients with AUD [12–19]. In patients with AUD, slow waves, including delta or theta waves, are elevated in the frontal lobe, which deteriorates frontal lobe functions; additionally, there are characteristic beta and alpha wave changes. Compared with healthy individuals, beta and alpha waves are increased and decreased, respectively, by more than three-fold. This suggests that patients with AUD have decreased cortical functions in regulating excitation and inhibition [20–24].

Previous QEEG studies on patients with AUD have primarily focused on the patients’ states rather than post-treatment changes, Moreover, no studies have conducted QEEG before and after an AUD intervention program to observe changes. Brain wave analysis is clinically significant since brain waves are strongly associated with AUD severity and recurrence [20,25–28]. In addition, biomarker studies may further elucidate the main mechanisms underlying AUD.

### AUD and the Minnesota Multiphasic Personality Inventory-2 (MMPI-2)

In the past decades, personality traits have been identified as important predictors of treatment success and relapse in patients with AUD [29–31]. Patients with AUD usually present with a lack of behavioral control, including aggression, impulsivity, and sensation seeking, as well as negative emotions, including depression and anxiety. Furthermore, patients with AUD exhibit a lack of insight, immaturity, irresponsibility, low motivation for change, and reduced environmental adaptability [32]; additionally, AUD is significantly correlated with an increased score for antisocial tendencies score in the MMPI-2 [33–35]. According to Yoon et al. [36] personality traits, including emotional traits, in patients with AUD remain relatively constant throughout their lifetime regardless of aging. Since an extended therapeutic intervention may not induce marked changes in drinking habits and behaviors, the patients’ personality traits, including aggression and impulsivity, and emotional aspects, including depression and anxiety, should be considered in the treatment interventions. Although the MMPI-2 remains a standard clinical tool, there is limited evidence regarding the therapeutic utility of this diagnostic approach, i.e., the degree of improvement of treatment outcomes attributable to the diagnostic approach [37,38].

### AUD and AT

According to the American Art Therapy Association, AT facilitated by a professional art therapist can effectively support personal and relational treatment goals as well as community concerns [39]. AT has been used to treat addiction since the 1950s, with extensive reports regarding its benefits. These reports have reduced apprehension regarding alcoholism treatment [5] and have provided a means of communication [40,41], with several benefits to patients [42]. In AT, working with creative ideas can facilitate the first step of treatment, as it can remove psychological resistance in patients with AUD and facilitate acceptance of the disease [43,44]. AT can accelerate personal growth in patients with AUD showing low self-efficacy by encouraging them to reflect on internal conflicts, express themselves, and divert undesirable impulses through creative activities [45]. Patients with AUD can improve self-confidence issues within interpersonal relationships through group AT [46]. In group AT, group members naturally interact with each other. Specifically, they exchange and acquire new information, behavioral cues, thought processes, and emotions that they imitate and act out. Group members can effortlessly bond as they mitigate their distorted emotions and thoughts [47]. Patients with AUD are often impulsive, selfish, anti-social, and dependent given their low threshold to endure tension [48]. However, AT can relieve depression and anxiety in these patients through creative self-expression and the acquisition of self-managing skills [49].

Since art can only function following the laws of the visual brain, patients with AUD can be trained to regulate the loss of control and impulsive urges resulting from brain damage [50]. Hass-Cohen and Findlay reported that artistic experience can enhance emotional regulation and cognitive function by regulating neurocognitive processes [51]. Furthermore, AT can stimulate the central nervous system to trigger or change a specific emotion and facilitate translation of the emotional and physiological responses to the stimulation into behavior. Therefore, artwork created in AT can be considered as an outcome of an individual’s neurophysiological pathway [52,53]. These outcomes resulting from integrating various emotional experiences through perception into the treatment process distinguish AT from other psychological therapies.

Regarding AT-related brain waves, Belkofer and Konopka reported a marked increase in alpha and beta activities after painting and drawing [54]. SimilarlyBelkofer et al. [55] reported altered alpha activity after drawing in both artists and non-artists. This suggests that AT increases relaxation linked to alpha waves. MoreoverKaimal et al. [56] observed activation of the frontal cortex during coloring, doodling, and free drawing on functional near-infrared spectroscopy. Therefore, AT may facilitate specified and aggressive treatment of patients with AUD.

We aimed to investigate the effect of AT on emotional (Minnesota Multiphasic Personality Inventory-2 [MMPI-2], alcohol dependence, depression, anxiety, and impulsivity) and physical (natural killer [NK] cell count, expression of stress-associated proteins [SAP], and electroencephalography) changes in patients with AUD.

## Materials and methods

### Participants and procedure

The study was conducted from March 2017 to March 2018 at KARF St. Mary’s Hospital in Ilsan, Republic of Korea. We recruited 55 patients with AUD; all met the study criteria and provided informed consent. We randomly allocated the 55 participants into the control (n = 27) and experimental (n = 28) groups. However, 20 participants dropped out due to recurrence of alcohol use. Finally, 15 and 20 participants from the control and experimental groups, respectively, completed the study.

### Randomisation method and allocation concealment

**Fig 1:**
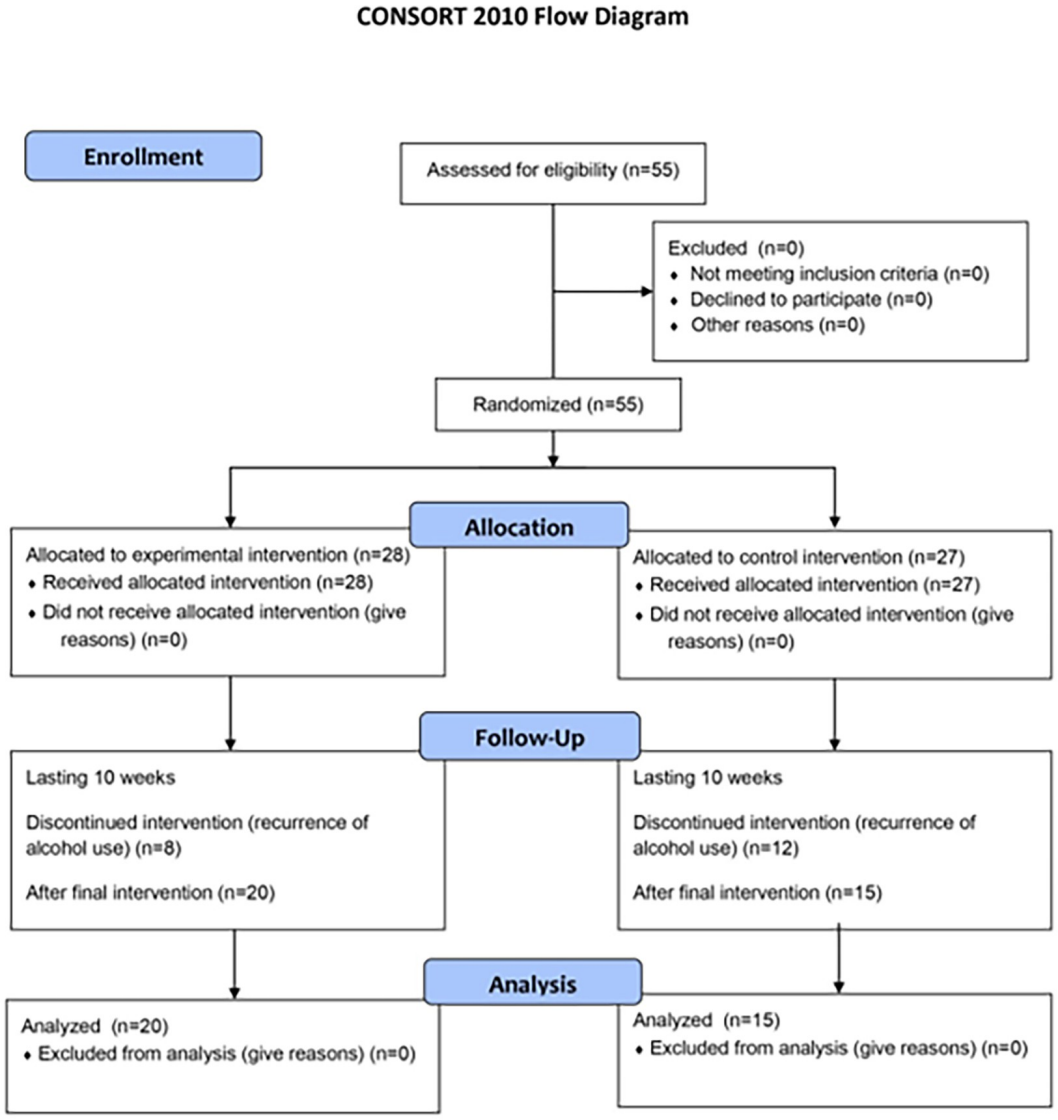
Recruitment and randomized allocation group from the CONSORT 2010 flow diagram.

**Fig 2:**
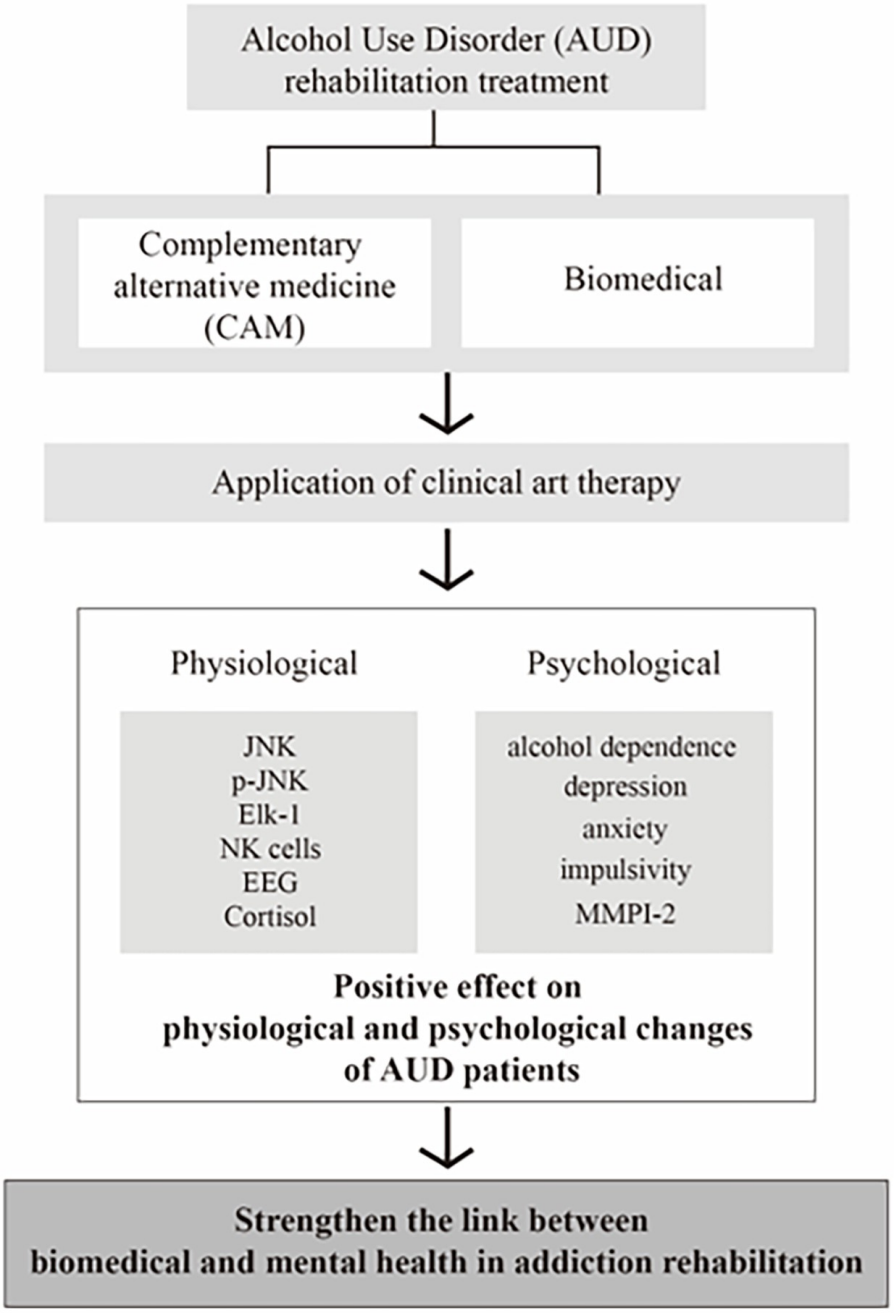
The overall flow of research.

This study was a single-blind study; the hospital’s clinical pathologists and doctors in contact with the patients were blinded, while the clinical art therapists conducting the study were not blinded. Participants were randomly assigned to groups using a computerized random number table by university volunteers who did not participate in the study intervention. The research description and informed consent form explained that the probability of being assigned to each group is 50:50. The assignment order was hidden in sequentially numbered, opaque, sealed, and stapled envelopes that did not transmit intense light in which participants were enrolled. Participants’ names and dates of birth were recorded on the envelopes to prevent the dispensing order from being reversed. Results were available after enrolled participants had completed all baseline assessments and before assigning interventions. Both groups received the same hospital-provided addiction treatment (AUD education, cognitive behavioral therapy, anger control training, recurrence prevention training, high-risk situation management program, etc.) (Figs 1 and 2).

We observed the changes in NK cell number and SAP expression, emotion, and behavior by applying AT, a complementary alternative medicine (CAM) to the psychological risk factors of AUD. Resulting changes in psychological factors led to positive NK cell growth and changes in SAP expression. It has also been shown that positive changes in the MMPI-2 profile can also change the personality of the individual. This shows the connection between physiology and mental health in addiction rehabilitation and shows the importance of psychological rehabilitation. This study was the first to show the biological basis of the application of complementary therapies, a non-drug treatment method of AUD, to humans and to monitor progress.

### Participants

The inclusion criteria were as follows:

- Adult patients diagnosed with AUD by a psychiatrist.
- Capable to participate in AT according to a psychiatrist
- Hospitalized in the research facility with completion of the 2-week detox
- Capability to perform minimal motor activities required for AT.
- Having received consent from their guardians to participate in the study.

The exclusion criteria were as follows:

- Pregnant or lactating during the trial
- Voluntary termination of the study.
- Difficulty in undergoing further studies due to serious diseases
- Patients who did not meet the subject criteria and participated falsely or caused physical and mental damage due to serious self-harm.

### Sample size

This study was a pilot study. Per Sheatsley and Sudman, a pilot study was appropriate with a minimum of 12 to 50 participants prior to a full-scale study [57,58]. This was a cost-, energy-, and time-efficient number of people while still being large enough. In addition, Moore et al. [59] recommended a minimum of 12 participants per group when designing large studies. In this study, the sample sizes of pilot studies were referenced and applied.

### Ethical approval

This study was conducted in accordance with the Declaration of Helsinki. Ethical approval was obtained from the CHA University Institutional Review Board in March 2017 (1044308-201612-BR-030-03) (S1 and S2 Files).

The clinical trial registration number is as follows:

Clinical Research Information Service [Internet]; Osong (Chungcheongbuk-do): Korea Centers for Disease Control and Prevention, Ministry of Health and Welfare (Republic of Korea); KCT0004588; December 31, 2019

### Informed consent

All the participants provided written informed consent. The consent form outlined the purpose, process, method, duration, side effects or risks, benefits, and disadvantages of the study. Additionally, the confidentiality of the collected personal information was guaranteed.

### Intervention

A clinical art therapist and two assistant therapists conducted the AT program. The main therapist was an AT expert with over 6 years of experience. The assistant therapists were graduate students in their master’s courses in clinical AT. The assistant therapists explained how to use the materials and helped participants with difficulty expressing their work. Pre- and post-test results, photographs of the work, and consultations were recorded after completion of the program.

Only the experimental group underwent AT, which involved weekly 60–90 min group therapy sessions conducted for 10 weeks. Both groups underwent the same post-test 10 weeks after the pretest. AT was administered as group therapy, with each group comprising nine patients. The group therapy was performed in a sufficiently large lecture hall at the study facility. For the first 15 min of the session, the therapist explained the session’s topic; further, the participants talked about recent events about themselves. Subsequently, the participants chose materials and expressed the topic in their art for 40 min. They were allowed to freely communicate during this process; moreover, they concentrated on their work. After completing their artwork, the participants and therapist discussed the drawings of each member for 10–30 min.

AT was conducted as part of the intervention program for AUD, which was structured according to the psychological problems of AUD. Table 1 presents details regarding the program used in this study.

**Table 1:** AT intervention program.

Session	Program	Activities	Expectancy effect
1	Rainbow of My Life	Depict the happiest moment of your life in drawing.	Introspection, finding trigger of alcoholism
2	What does alcohol mean to me?	Explore what alcohol means to you and express it directly.	Introspection, investigating cause of drinking, recalibration of health drinking habits
3	Mandala	Reflect and concentrate on the inner workings of your mind.	Recognition and examination of emotions currently felt
4	Reconstructing Masterpieces	Reconstruct a famous painting of your choice.	Recognition of inner self and reflection on personal image.
5	Self-portrait	Represent how you see yourself and how other people see you with using clay.	Recognition of true self and externally projected self
6	Bankbook of My Mind	Reflect on what thoughts are in my mind and depict them in pictures.	Enhancement of emotional awareness and control, self-control
7	Bag of Promises	Make a bag of promises with construction paper and write down the promises you wish to make with yourself and your family.	Regaining family-life balance, autonomy, understanding emotional anguish felt by family, enhancing self-regulation
8	Tree that grows with family (friends)	Draw a tree representing yourself, then draw families and friends who nourish you as its roots.	Looking for ways to restore damaged relationships, establishing balanced relationship, self-esteem
9	Myself I aspire (I no longer dependent on alcohol)	Imagine and draw your changed self.	Discovering positive aspects of oneself, forming positive self-image, reducing emotional dependence
10	Puzzle of My Life	Collect and gather small pictures to create a larger picture.	Establishing balanced relationship, improving self-esteem

## Measures

### Detection of changes in the immune cell count and SAP expression levels

A blood test for SAPs allows objective assessment of the positive effect of AT on the immune system and stress levels. We tested for cortisol levels, NK cell count, and SAP expression levels (C-Jun N-terminal kinase [JNK], p-JNK, and Elk-1).

Elk-1 is a transcription factor that directly regulates the expression of immediate early genes and is crucially involved in long-term memory, drug addiction, Alzheimer’s disease, Down’s syndrome, breast cancer, and depression [60–62]. JNK and p38 cascades respond to cytokines and stress [63]. JNK is involved in the occurrence of various illnesses; moreover, it is activated by protein synthesis inhibitors and other stress stimuli [64]. JNK activates c-Jun, ATF2, ELK-1, SMAD4, p53, and HSF1; further, by activating and inhibiting other small molecules, JNKs regulate important cellular functions, including growth and differentiation [63].

### Electroencephalography (EEG)

EEG was performed using the Neurofeedback System (Panaxtox Corp, Seoul, Korea) developed by the Korea Psychiatry Research Center. This device simultaneously measures the left and right EEG traces in FP1 and FP2 of the prefrontal lobe using two electrodes in a sequential bipolar montage, following the International 10–20 system. A solid electrode was attached to the headband; subsequently, EEG was measured from the left and right frontal lobes through the FP1, FPz, and FP2 channels, which were fixed at 4-cm intervals on the left ear lobe as a round electrode. EEG measurements assess real-time brain function and allow objective brain function analysis. EEG is a frequency-based spectrum analysis method that captures the degree of slow and fast waves through correlation and provides more information than existing analysis methods for each [65]. Therefore, the brain function index can be ascertained through EEG analysis (Table 2).

**Table 2:** The brain function quotient.

Analyzing Quotient	Hemisphere	Related Frequency	Characteristics
Attention quotient (ATQ)	Left, right	Theta (θ) wave SMR	Decision of brain awakening degree, resistance to disease or physical fatigue
Activity quotient (ACQ)	Left, right	Alpha (α) wave Low beta (β) wave	Decision the active function of the brain

#### Attention quotient (ATQ)

ATQ represents the degree of brain arousal and resistance to disease or stress. It is calculated by dividing theta wave activity by sensorimotor rhythm (SMR) wave activity of approximately 12–15 Hz. Therefore, it is related to immunity against diseases or stress [66,67].

#### Activity quotient (ACQ)

The ACQ is an index representing the level of alpha and low-beta wave activity as well as the overall activity in the left and right brain. It is used to determine mental activity, thinking, and behavioral tendencies. It can be computed by analyzing alpha and low-beta waves. High and balanced ACQs between the left and right brains are ideal [66,67].

### Minnesota multiphasic personality inventory-2 (MMPI-2)

The MMPI-2 is a widely used self-reporting test developed by Hathaway and McKinely for accurate clinical diagnosis and evaluation of patients with psychiatric disorders [68]. Currently, it is used to objectively assess patients’ symptoms, personality traits that may affect psychopathology, adaptive performance, and attitudes toward testing.

### Alcoholism screening test of the National Seoul Mental Hospital (NAST)

The NAST was developed by Kim et al. [69] for AUD diagnosis. A NAST score ≥ 11 suggests an advanced stage of AUD.

### Center of Epidemiologic Studies Depression Scale (CES-D)

The CES-D was developed by the National Institute of Mental Health in 1971 to measure depressive symptomatology in the general population. We used the Korean version translated by Cho and Kim [70].

### Beck anxiety inventory (BAI)

Anxiety was measured using the BAI, which is a self-report questionnaire developed by Beck, Emery, & Greenberg. It allows for the assessment of clinical anxiety in patients with psychiatric disorders and comprises 21 items regarding cognitive, emotional, and physical anxiety [71].

### Barratt impulsiveness scale-II (BIS-II)

We used a modified version of the 11^th^ edition of the 35-item BIS-II [72] developed by Lee. Higher scores indicate greater impulsivity in patients [73].

### Data collection and procedures

Both groups underwent baseline assessment before the first session and post-intervention assessment after the final session. The EEG and questionnaire surveys were conducted in a testing room in the study facility without disturbances. Moreover, laboratory tests for the NK cell count, cortisol levels, and protein levels were conducted by a clinical pathologist at the study facility. The EEG was performed within 10 min, including preparation, while the survey, including the MMPI-2, took about 90–120 min. Participants were assigned an identification number, which was used to manage and document their baseline and post-intervention data.

#### Statistical analysis

Statistical analyses were performed using SPSS Statistics (version 22.0). Specifically, we performed a frequency analysis of the participants’ sociodemographic characteristics. Although normality was assumed through the normality test, a nonparametric test was performed because the number of study participants was small, and an ordinal scale was used. Ranked ANCOVA was performed for the NK cell count and cortisol levels. The MMPI-2, SAP, EEG, depression, anxiety, and impulsivity data were analyzed using Wilcoxon’s signed rank test. Statistical significance was set at *a* = 0.05.

#### Western blotting

SAP expression was measured using serum samples obtained from both groups. Bradford assay was performed to measure and quantify protein levels in each sample, followed by protein analysis using Western blotting with anti-JNK (Cat# SC-571 Santa Cruz, Santa Cruz, CA, USA), anti-p-JNK (Cat# SC-571 Santa Cruz, Santa Cruz, CA, USA), and anti-Elk-1 antibodies (Cat# SC-355 Santa Cruz, Santa Cruz, CA, USA). First, the total protein concentration in each serum sample was quantified using the Bradford method. Accordingly, 20 μg of protein was analyzed through sodium dodecyl sulfate-polyacrylamide gel electrophoresis at 120 volts for 1.5 h.

Subsequently, proteins in the gel were transferred to a nitrocellulose membrane (Bio-Rad, CA, USA) at 120 Volts for 1 h. The membranes were blocked for 30 min at room temperature with 5% blocking buffer, which was prepared by dissolving 2.5 mg of nonfat milk in 50 ml of washing buffer comprising 10 mM (5 ml of 2M stock) Tris-HCl (pH 7.5), 100 mM (20 ml of 5M stock) NaCl, 1 ml of 0.1% Tween 20, and 976 ml distilled water. The membranes were probed with primary antibodies against SAP, p-SAP, and Elk-1 (all 1:1,000 dilution), followed by incubation at 4°C overnight. After washing with washing buffer, the membranes were incubated for 1 h with horseradish peroxidase-conjugated polyclonal goat anti-rabbit IgG (1:10,000 dilution). The membranes were re-washed with washing buffer and developed on an X-ray film with ECL reagents (enhanced chemiluminescence kit, Youngin Frontier, Seoul, Korea).

## Results

We randomly allocated the 55 participants into the control (n = 27) and experimental (n = 28) groups. However, 20 participants dropped out due to recurrence of alcohol use. Finally, 15 and 20 participants from the control and experimental groups, respectively, completed the study. With the exception of dropouts, all participants proceeded as they were in their initially assigned group. The average age of the samples analyzed for the control and the experimental groups was 46.9 years (SD = 11.7161) and 40.7 years (SD = 8.8858), respectively. The alcohol dependence screening test revealed that most participants were at an advanced AUD stage, with 34.3% and 85.8% scoring above 30.1 and 11, respectively (Table 3).

**Table 3:** General characteristics of the control and experimental groups.

Classifiation		Control group (n = 15)	Experimental group (n = 20)
		N (%)	N (%)
Gender	Male	12 (80)	16 (80)
	Female	3 (20)	4 (20)
Age	20–29 years old	1 (6.7)	0 (0.0)
	30–39 years old	3 (20.0)	11 (55.0)
	40–49 years old	6 (40.0)	6 (30.0)
	Over 50 years old	5 (33.3)	3 (15.0)
Marital status	married	4 (26.7)	7 (35)
	single	7 (46.7)	11 (55)
	divorce	2 (13.3)	2 (10)
	separation	1 (6.7)	0 (0)
	Etc	1 (6.7)	0 (0)
Hospitalization experience	No hospitalization	1 (6.7)	0 (0)
	1 time	3 (20)	10 (50)
	2 times	2 (13.3)	4 (20)
	3 times	1 (6.7)	2 (10)
	4 times	3 (20)	0 (0)
	5 times	1 (6.7)	2 (10)
	6 times	0 (0)	2 (10)
	7 times	1 (6.7)	0 (0)
	More than 10 times	3 (20)	0 (0)
3 months experienced abstinence	Yes	10 (66.7)	13 (65.0)
	None	5 (33.3)	7 (35.0)
Family history of drinking	Yes	7 (46.7)	14 (70.0)
	None	8 (53.3)	6 (30.0)
Family drinking experience	Very unlikely	0 (0)	1 (5.0)
	Unlikely	2 (13.3)	2 (10.0)
	Normal	3 (20.0)	1 (5.0)
	Likely	6 (49.0)	8 (40.0)
	Very likely	4 (26.7)	8 (40.0)
Drinking people around	Very unlikely	0 (0.0)	1 (5.0)
	Unlikely	1 (6.7)	1 (5.0)
	Normal	1 (6.7)	2 (10.0)
	Likely	7 (46.6)	10 (50.0)
	Very likely	6 (40.0)	5 (25.0)
Family disease acceptance	Very unlikely	2 (13.3)	3 (15.0)
	Unlikely	2 (13.3)	2 (10.0)
	Normal	4 (26.7)	2 (10.0)
	Likely	5 (33.3)	5 (25.0)
	Very likely	2 (13.3)	8 (40.0)
NAST score (Total score 35.2)	0–10.9	1 (6.7)	4 (20.0)
	11–20	3 (20.0)	5 (25.0)
	20.1–30	5 (33.3)	5 (25.0)
	30.1–35.2	6 (40.0)	6 (30.0)

### Group comparisons

#### Changes in the NK cell count, cortisol levels

To determine the effectiveness of AT, Ranked ANCOVA was performed to compare experimental and the control groups in the change scores of NK cell, and Cortisol. The results of Ranked ANCOVA is presented in Table 4. As shown in Table 4, compared with the control group, the experimental group showed an increase in the NK cell count (*F* = 8.285, *p* = 0.007). Additionally, there was a decrease in cortisol levels after AT; however, there was no significant between-group difference (*F* = 0.000, *p* = 0.997).

**Table 4:** Ranked analysis of covariance for NK cells, and cortisol.

Classification	Control group (*n* = 15) *M* (*SD)*		Experimental group (*n* = 20) *M (SD)*		*F*	*p*	eta-squared
	Before	After	Before	After			
NK cell	13.527 (8.1119)	11.507 (7.6979)	13.270 (5.7493)	15.495 (5.5590)	8.285**	0.007	0.1
Cortisol	11.007 (3.4964)	11.333 (4.5140)	12.235 (4.3993)	11.650 (4.4615)	0.000	0.997	0.0

** *p <*0.01.

#### Changes in stress-associated proteins

**Fig 3:**
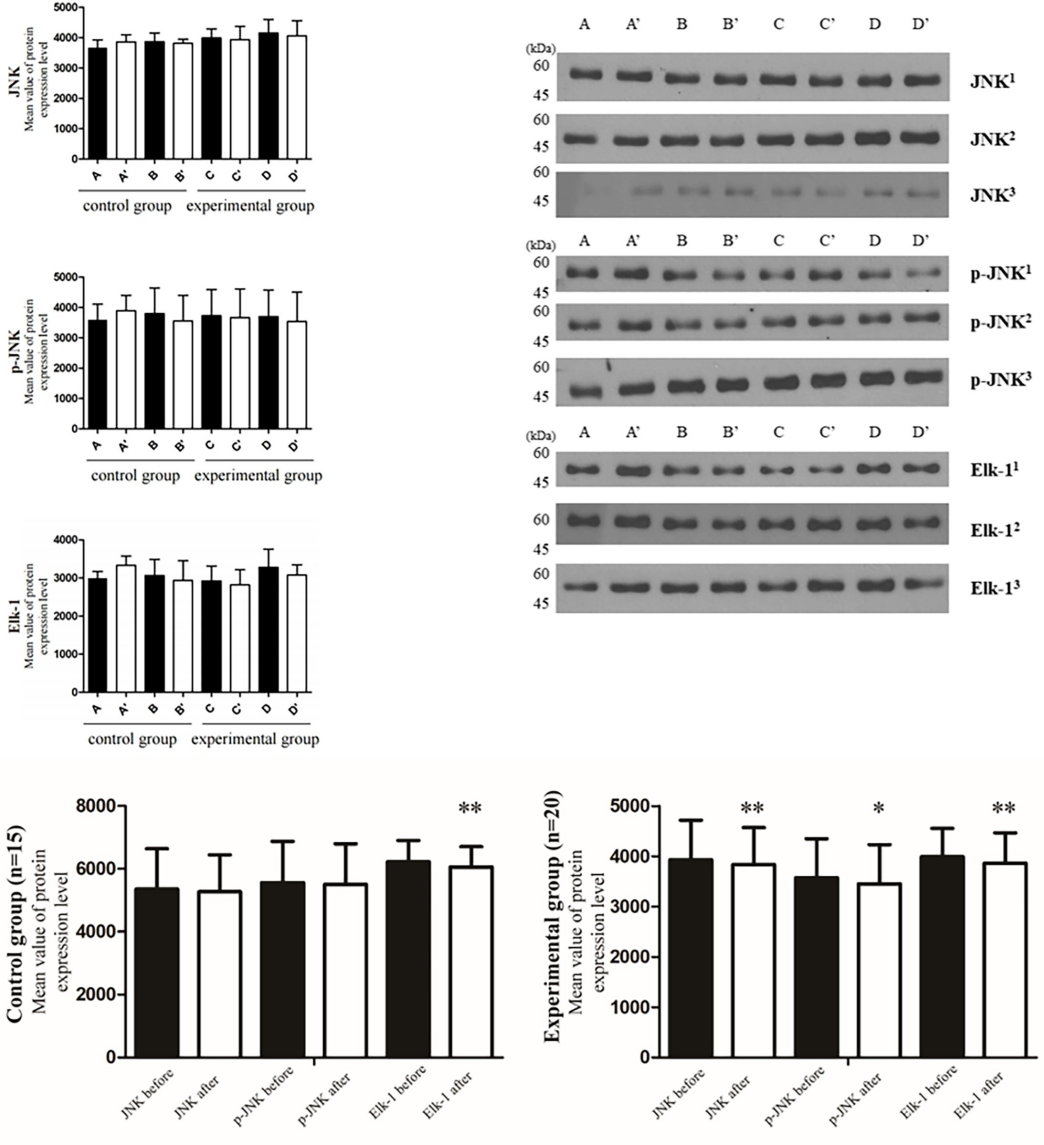
Changes in participants’ SAP expression level before and after AT. **(A, B)** Western blotting repeated experiments were conducted on four randomly selected participants in the control and experimental groups.
Control group pre- test: A, BControl group post- test: A’, B’Experimental group pre- test: C, DExperimental group post- test: C’, D’. **(C)** Results of Wilcoxon’s signed-rank test of Western blotting results of the control group (n = 15), and experimental group (n = 20) Control group: JNK (Z = -1.022, *p* = 0.307), p-JNK (Z = -1.079, *p* = 0.281), and Elk-1 (Z = -2.613, *p* = 0.009) Experimental group: JNK (Z = -2.613, *p* = 0.009), p-JNK (Z = -2.501, *p* = 0.012), and Elk-1 (Z = -2.651, *p* = 0.008). ** *p <*0.01, * *p <*0.05.

We analyzed changes in SAP expression in both groups. Repeated western blotting experiments were performed on two randomly selected participants from each group (Figs 3A, 3B and S1). Based on these results, western blotting was performed for all participants (Fig 3C and Table 5). There were significant between-group differences in the levels of JNK (Z = -2.613, *p* = 0.009), p-JNK (Z = -2.501, *p* = 0.012), and Elk-1 (Z = -2.651, *p* = 0.008). The control group showed non-significant changes in SAP expression.

**Table 5:** Means and Wilcoxon’s signed-rank test comparisons for study variables.

Classification		Control group (*n* = 15) *M (SD)*				Experimental group (*n* = 20) *M (SD)*				Effect size
		Before	After	*Z*	*p*	Before	After	*Z*	*p*	*d*
ATQ	Left	57.95 (11.05)	58.45 (11.36)	-0.057	0.955	52.14 (11.05)	58.73 (11.36)	-2.222*	0.026	0.47
	Right	60.03 (9.13)	58.91 (11.70)	-0.852	0.394	54.40 (9.13)	61.15 (11.70)	-2.632**	0.008	0.71
ACQ	Left	67.71 (14.39)	69.36 (15.78)	-0.596	0.551	58.39 (12.09)	64.50 (14.46)	-2.576*	0.010	0.42
	Right	67.95 (15.249)	69.95 (17.29)	-0.795	0.427	60.14 (11.08)	65.17 (12.52)	-2.053*	0.040	0.30
Stress proteins	JNK	5370.25 (1228.00)	5270.95 (1169.67)	-1.022	0.307	3926.01 (787.08)	3832.82 (735.92)	-2.613**	0.009	0.02
	p-JNK	5565.84 (1303.56)	5502.38 (1295.39)	-1.079	0.281	3572.05 (774.39)	3453.32 (774.39)	-2.501*	0.012	0.26
	ELK1	6222.01 (671.66)	6056.47 (648.92)	-2.613**	0.009	3988.62 (656.27)	3858.69 (604.87)	-2.651**	0.008	0.17
NAST		25.30 (9.05)	26.81 (10.46)	-1.260	0.208	22.66 (10.47)	10.81 (9.02)	-3.920***	0.000	1.73
CED-D		27.20 (13.41)	27.867 (14.1566)	-0.534	0.593	28.00 (14.88)	10.10 (8.87)	-3.824***	0.000	1.50
BAI		14.40 (11.11)	16.333 (9.54)	-1.170	0.242	20.40 (16.02)	7.850 (7.46)	-3.466**	0.001	1.12
BIS- Ⅱ		18.13 (8.26)	19.600 (7.11)	-1.828	0.068	17.70 (8.27)	12.950 (7.27)	-2.965**	0.003	1.12

*** *p <*0.001.

** *p <*0.01.

* *p <*0.05.

Effect size cut offs: 0.20 small, 0.50 medium, ≥ 0.80 large.

ATQ, attention quotient; ACQ, Activity quotient.; Stress proteins (JNK, p-JNK, ELK1); NAST, Alcoholism screening test of the National Seoul Mental Hospital; CED-D, Center of Epidemiologic Studies Depression Scale; BAI, Beck anxiety inventory; BIS- Ⅱ, Barratt impulsiveness scale-II.

#### Changes in the EEG, NAST, impulsivity, depression, and anxiety

As shown in Table 5, AT positively affected changes in brain waves. Specifically, the experimental group showed positive ATQ [left (Z = −2.222, *p* = 0.026) and right (Z = −2.632, *p* = 0.008)] and ACQ changes [left (Z = −2.576, *p* = 0.010) and right (Z = −2.053, *p* = 0.040)]. However, there were no significant changes in the control group.

In addition, the experimental group decreased alcohol dependence (Z = −3.920, *p* = 0.000), impulsivity (Z = −2.965, *p* = 0.003), anxiety (Z = −3.466, *p* = 0.001), and depression (Z = −3.824, *p* = 0.000) compared to the control group, and showed statistically significant changes.

### Changes in the MMPI-2 profile

The experimental group showed significant changes in the validity scale of the MMPI-2 (F, F (B), L, K, and S) (Table 6). This indicates increased defensiveness; however, it can be interpreted as an attempt to project a better version of oneself or perceiving one’s situation as better than it is. The distribution of the mean MMPI-2 score was within the range of valid data. There was no significant score change in the control group, indicating no change in self-perception.

**Table 6:** Means and Wilcoxon’s signed-rank comparisons for MMPI-2 (validity scales and clinical scales).

Classification	Control group (*n* = 15) *M (SD)*				Experimental group (*n* = 20) *M (SD)*				Effect size
Classification	Before	After	*Z*	*p*	Before	After	*Z*	*p*	*d*
VRIN	47.600 (7.5951)	45.533 (11.8011)	-1.007	0.314	43.950 (7.2582)	43.150 (6.9151)	-0.856	0.392	0.12
TRIN	56.067 (4.6975)	58.200 (7.7201)	-1.198	0.231	57.550 (6.0217)	58.200 (7.4452)	-0.223	0.824	0.22
F	52.467 (8.7003)	50.533 (8.3910)	-1.025	0.305	53.150 (10.4240)	47.100 (7.2250)	-2.896**	0.004	0.53
Fb	52.200 (11.7850)	50.933 (9.7429)	-0.541	0.589	51.650 (9.7941)	46.500 (7.3878)	-2.705**	0.007	0.50
Fp	50.200 (7.6270)	48.133 (6.6748)	-1.266	0.206	45.45 (5.8802)	46.500 (4.8612)	-1.234	0.217	0.60
L	49.800 (9.8213)	51.467 (10.9601)	-0.717	0.473	46.550 (10.6251)	52.800 (12.3357)	-2.559*	0.010	0.51
K	50.000 (10.7305)	51.667 (13.2270)	-0.892	0.373	48.850 (10.0957)	52.700 (11.8504)	-2.338*	0.019	0.34
S	46.800 (10.3662)	50.400 (12.9318)	-1.070	0.284	45.800 (12.7882)	52.550 (12.9268)	-3.138**	0.002	0.34
Hs	52.267 (10.0887)	51.400 (7.5290)	-0.157	0.875	54.750 (11.1160)	51.550 (11.7495)	-1.471	0.141	0.28
D	55.733 (11.8651)	55.000 (11.3137)	-0.350	0.727	58.950 (11.4454)	53.000 (10.4579)	-2.821**	0.005	0.49
Hy	54.267 (11.4920)	55.400 (11.2301)	-1.140	0.254	55.550 (8.6358)	52.650 (8.3368)	-1.329	0.184	0.43
Pd	63.400 (10.2595)	59.867 (8.1404)	-1.289	0.197	65.750 (13.0056)	58.750 (12.4388)	-2.376*	0.018	0.31
Mf	51.467 (9.0306)	50.933 (9.4979)	-0.314	0.753	52.650 (10.6993)	51.900 (11.2713)	-0.475	0.635	0.02
Pa	56.733 (11.0613)	54.200 (10.7383)	-1.022	0.307	55.500 (11.5280)	49.650 (8.1968)	-2.251*	0.024	0.32
Pt	54.067 (11.9610)	53.733 (15.3691)	-0.126	0.900	58.700 (15.2319)	50.250 (12.9325)	-2.934**	0.003	0.79
Sc	53.600 (11.9630)	51.867 (11.2050)	-0.943	0.346	54.750 (14.5670)	48.700 (10.7610)	-2.357*	0.018	0.43
Ma	52.867 (11.8856)	51.200 (14.8093)	-0.094	0.925	50.150 (11.3660)	47.850 (13.5657)	-1.232	0.218	0.06
Si	45.067 (1.1028)	46.200 (10.6046)	-0.789	0.430	55.650 (15.6685)	50.200 (14.3182)	-2.855**	0.004	0.99

** *p <*0.01

* *p <*0.05

Effect size cut offs: 0.20 small, 0.50 medium, ≥ 0.80 large.

VRIN (Variable Response Inconsistency), TRIN (True Response Inconsistency), F (Infrequency), F(B) (Back Infrequency), F(P) (Infrequency Psychopathology), FBS (Symptom Validity), L (Lie), K (Correction), S (Superlative Self-Presentation), Hs (Hypochondriasis), D (Depression), Hy (Hysteria), Pd (Psychopathic Deviate), Mf (Masculinity-Femininity), Pa (Paranoia), Pt (Psychasthenia), Sc (Schizophrenia), Ma (Hypomania), Si (Social Introversion).

Moreover, the experimental group showed significant changes in the clinical scale (D, Pd, Pa, Pt, Sc, and Si), indicating a positive change in the levels of depression, antisocial tendencies, paranoia, obsessive-compulsive disorder, schizophrenia, and introversion.

There was a significant change in the levels of demoralizing negative emotions, cynical attitudes, dysfunctional negative emotions, and aberrant experiences in the experimental, but not control, group (RCd, RC2, RC3, RC7, and RC8) (S1 Table).

In the personality pathology 5 factor scale, the experimental group showed a significant change in negative emotionality and introversion AT (S1 Table). Moreover, there was a significant change in negative emotion, neurosis, introversion, and low positive emotions in the experimental, but not control, group. This suggests that the experimental group showed improved positive emotions and decreased negative emotions. Furthermore, the improved interpersonal relationship increased others’ consideration and produced more stable interpersonal relationships. This further indicates improved self-esteem and self-reflecting emotions. The experimental group showed significant changes in anxiety, depression, bizarre mentation, aberrant state, cynical attitude, type A behavior, self-esteem, family problems, occupational difficulties, negative treatment indices, self-intensity, college life maladjustment, post-traumatic stress disorder, marital maladjustment, and addiction recognition (S2 and S3 Tables).

Twenty patients dropped out of this study, which indicates the difficulty of rehabilitation in addiction treatment and the high recurrence rate. There was a significant change in the DISC score in the control group since it had lower pre-test scores than the experimental group. Since a considerable number of patients in the control group dropped out, those who did not could have been able to cope with the possibility of controlling themselves and had a low impulsive tendency.

## Discussion

This study examined the physical, emotional, and behavioral changes experienced by patients with AUD after AT. First, we found that AT was associated with an increased NK cell count. Alcohol use decreases immune function; moreover, an increased NK cell count indicates increased immune function. The mechanisms through which socio-psychological factors affect immune function and the resulting physiological changes remain unclear [74]. However, our findings are consistent with previous reports that providing appropriate social support can strengthen the neuroendocrine and immune systems [75,76].

Second, the experimental group showed decreased JNK and p-JNK expression and variations in Elk-1 expression. These findings demonstrate that AT may alleviate stress and depression in patients with AUD. There was a significant between-group difference in SAP expression, which indicates an effect of AT on the body’s anti-inflammatory functions. Elk-1 is associated with drug addiction and depression, with activation by JNK. Future studies should investigate the involvement of Elk-1 in pathophysiological depression [64]. Alcohol use is a major risk factor for various diseases; therefore, there is a need to study JNK catalysis and its biochemical function within the body [63]. In the experimental group, there was a non-uniform reduction in Elk-1 levels due to within-group differences in the depression and immunity levels. Investigating psychotherapy-induced changes in Elk-1 may provide a scientific basis for the psychological effects in patients with AUD. Since patients with AUD often experience alcoholic dementia and cognitive decline, there have been studies on the role of Elk-1 in Alzheimer’s disease and long-term memory [77].

Third, there was a non-significant decrease and increase in cortisol levels in the experimental and control groups, respectively. Cortisol is crucially involved in regulating the limbic and immune systems; moreover, it is associated with changes in behavior, cognition, and immune responses [78]. Animal studies have indicated that stress suppresses the immune response by increasing blood cortisol levels [79]. However, there have been inconsistent clinical reports regarding whether increased cortisol levels accelerate or inhibit the immune response [79]. Decreased cortisol levels may be related to an increased NK cell count and could be indicative of behavioral and cognitive changes.

Fourth, AT caused positive changes in the brain waves of patients with AUD. The ATQ is calculated by dividing the theta wave activity by SMR wave activity (approximately 12–15 Hz) [66]. Theta waves are involved in encoding and retrieving working memory; moreover, they are crucially involved in peak performance. AT increased the number of alpha waves generated, which led to crossover with theta waves. SMR waves are closely related to concentration, which corresponds to the arousal preparation state or the standby state of the motor system [80,81]. These waves only appear in the sensory domain and are associated with immunity. The experimental group showed positive changes were observed in the left and right hemispheres. The positive changes in the ATQ suggest alterations of the prefrontal lobe functions such that patients have an increased tendency to proactively, rather than reflexively, respond to external stimuli in a premeditated manner. This can lead to positive physical changes.

The ACQ is determined by relative intensity, absolute intensity, log comparison, and arithmetic comparison [82,83]. A marked between-hemisphere imbalance in the value may increase the risk of brain functional imbalance, including emotional instability, behavioral instability, language impairment, and memory deterioration, which could cause morbidity [83]. Compared with the control group, the experimental group showed a significant increase in the mean ACQ for both hemispheres. The experimental group showed slightly more increased right brain activity, which indicates the strengthening of cognitive functions that coordinate responses to new situations as well as restructure and alter past experiences. Further, demonstrates that participants in the experimental group developed a good regulation of relaxation (alpha waves) and activation (beta waves).

The human brain has plasticity, where it consistently adapts and restructures based on learning and experiences [84]. Furthermore, the human brain uses five senses for perception and accepts stimuli, processes information, and responds accordingly. Accordingly, the brain accepts most incoming stimuli for information processing and serves as the main channel for delivering information. An emotion occurs through the activity of neurotransmitters that transmit emotion-indicating signals through synapses in brain areas, including neural circuits, that generate emotion. Thus, AT activities involving sensory stimulation from various media and one’s internal state positively affect changes and development of brain waves and structures by promoting neurogenesis and neural restructuring through comprehensive and reciprocal sensory experiences.

Fifth, the experimental group showed a reduction in depression, anxiety, aggression, impulsivity, alcohol dependence, and anti-sociality. The D scale, which showed the second-highest score in the MMPI-2 in our study, describes the likelihood of suicide attempts, sleep disturbances, vitality, and attention. These effects were markedly alleviated in the experimental group. Further, there were positive changes in DEP, RC7, and RC8, which can be attributed to decreased depression, anxiety, dysfunctional negative emotions, irritability, and aberrant experiences, as well as improved interpersonal relationships. The experimental group showed increased positive emotions and a decreased tendency to feel less positive emotions. There was a positive change in subjective well-being, which alleviated depression and anxiety. Moreover, the experimental group showed decreased aberrant sensory experiences and cynical attitudes. The relationship between AUD and depression is important because it may be a motivation for initial alcohol use. AT had a positive effect on the scores for psychasthenia and paranoia, indicating a relief effect on inappropriate feelings by controlling stress in patients with AUD who lack stress-coping strategies. Consistent with these findings, there was a significant between-group difference in the CES-D and BAI scores.

The experimental group showed significant improvement in the BIS-II score, as well as in the antisocial tendency and RC scales in the MMPI-2. Recent studies have shown that impulsivity increases the addiction frequency [85] and that cognitive impairment is associated with impaired emotional function, including identifying emotional facial expressions, in patients with AUD [86]. Both groups showed higher scores for antisocial tendencies and behaviors than in the other scales. This suggests that patients with AUD tend to rebel through antisocial and criminal behaviors. Moreover, it indicates that AT can mitigate various antisocial, criminal, and deviant behaviors, which are caused by problematic alcohol use. With the reduction in alcohol dependence, there were positive changes in scales related to interpersonal relationships. This could improve relationships within the family and mitigate negative thoughts about life; therefore, AT can be applied as a program for psychosocial rehabilitation.

Sixth, the experimental group showed improved social relations and self-concept. The MMPI-2, Si, and INTR scales could be associated with depression and social introversion, which causes difficulties in group settings and social life. There were significant changes in the schizophrenia, RC2, and RC3 scales in the experimental group, which indicated positive emotional involvement in life, decreased cynical attitudes, and social depression. AT could eliminate negative emotions through increased attention and acceptance of one’s emotions and others’ feelings, which causes positive changes in anxiety, inferiority, and depression. Group AT can help participants accept other people’s emotions.

### Limitations and suggestions for future research

This study had several limitations. First, we included a relatively small sample size. Given the high dropout rate of participants (due to relapse), there was a between-group difference in the sample size. This could undermine the generalizability of our findings. Second, we did not consider the influence of environmental factors, including the function of the participant’s family or differences in the surrounding environment. Third, we did not perform age- and gender-based comparisons. Finally, we only recruited participants from one research institute.

Future large-scale studies with gender-based comparisons are warranted to confirm the effect of AT. Additionally, there is a need for active prospective studies on the effects of psychological health on physical health, post-discharge abstinence, stress coping style, and networking with organizations. The home support system or other social factors can also influence the outcomes; therefore, an appropriate experimental design is required to control for such variables. A comprehensive outlook and approach to AUD are required from multidisciplinary, with the inclusion of both treatment and rehabilitation.

## Conclusions

The results of our study show that through AT, alcohol dependence, depression, anxiety, and impulsivity in patients with AUD decreased, and that AT was effective prevention and recovery. The results do not indicate a reduction in the number of patients with AUD but do suggest that AT is indeed beneficial in numerous ways.AT can be used as a stress control program for preventing relapse and can help patients with AUD become functional members of society. We found that psychological support through AT had a significant effect on immune response, stress response, and psychosocial measures. And by observing the changes, we found a link between psychological mechanisms and SAP. This study is the first of its kind to present biological evidence in the treatment of psychological problems that could have a physically negative impact on AUD patients in humans, and shows the connection between physiology and mental health in addiction rehabilitation and shows the importance of psychological rehabilitation. We hope that the results of this study would contribute to its applicability of AT as one of the intervention methods for treating AUD.

## Supporting information

S1 Checklist CONSORT 2010 checklist of information to include when reporting a randomised trial*. (DOC)

S1 Fig Original images for blots and gels. (PDF)

S1 Table Restructured clinical scales and Psy-5 scales T-scores were compared by the group. (DOCX)

S2 Table Content scales of T-scores were compared by the group. (DOCX)

S3 Table Supplementary scales of T-scores were compared by the group. (DOCX)

S1 File CHA University Institutional Review Board Form- English. (PDF)

S2 File CHA University Institutional Review Board Form- Korean. (PDF)
