# Supplementary figures and images for: A pilot randomized clinical trial of biomedical link with mental health in art therapy intervention programs for alcohol use disorder: Changes in NK cells, addiction biomarkers, electroencephalography, and MMPI-2 profiles
Source: PLoS One. 2023 May 5;18(5):e0284344. doi: 10.1371/journal.pone.0284344 (PMC10162529; doi:10.1371/journal.pone.0284344)

Original images for blots and gels

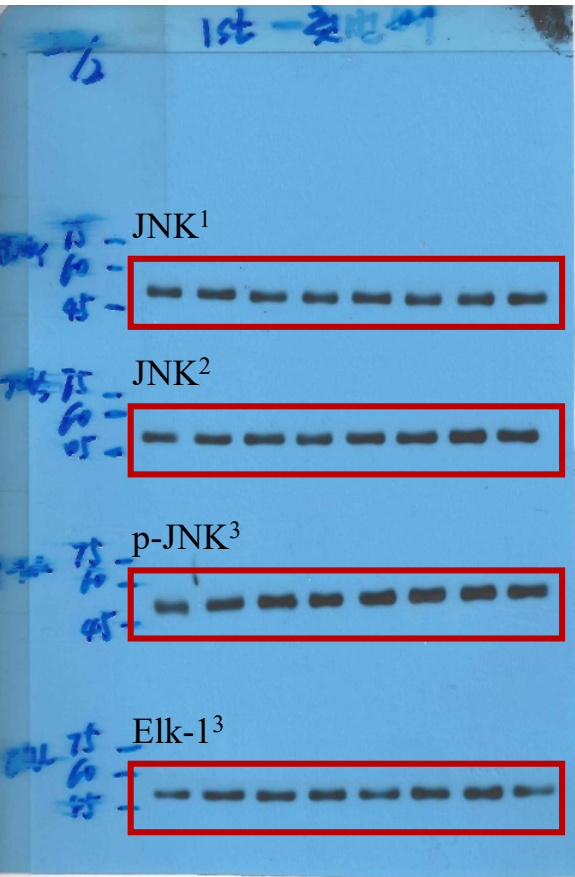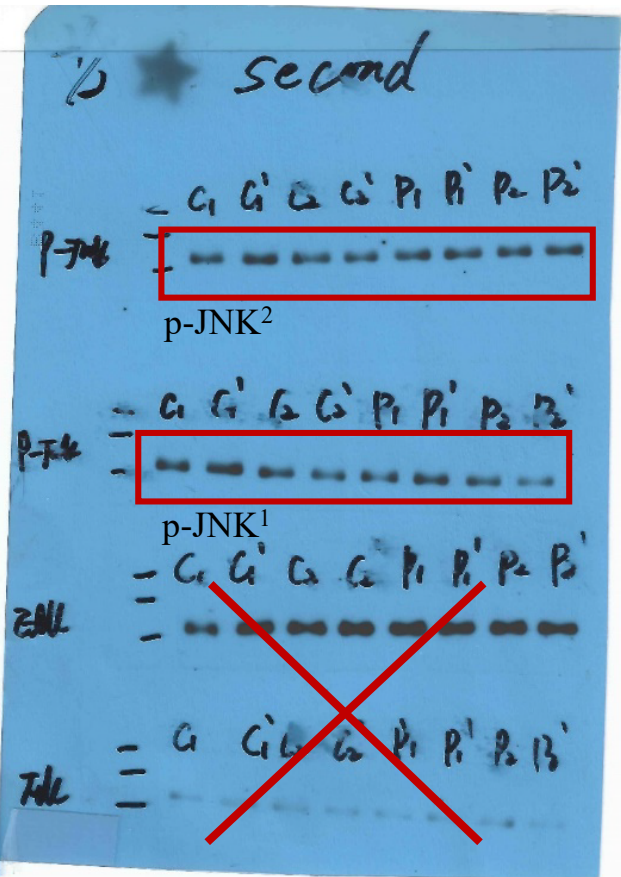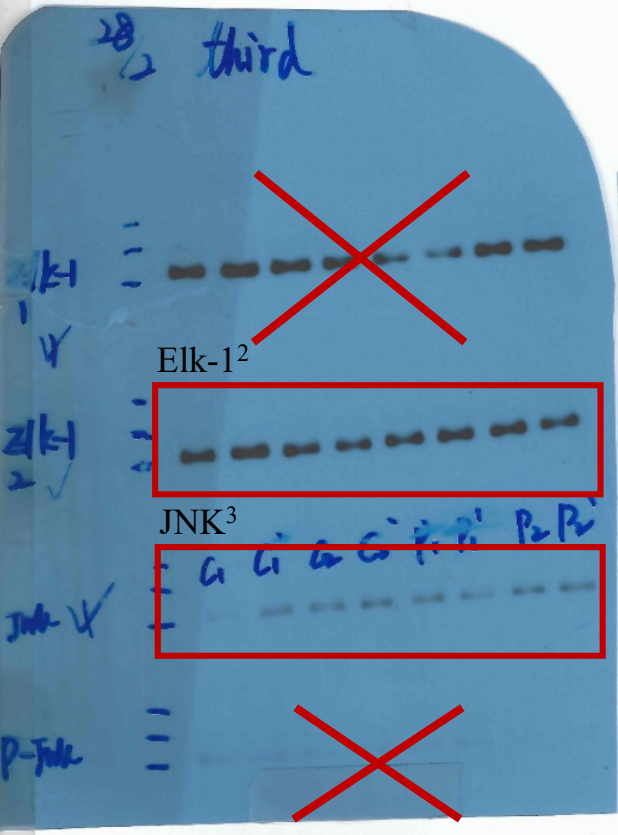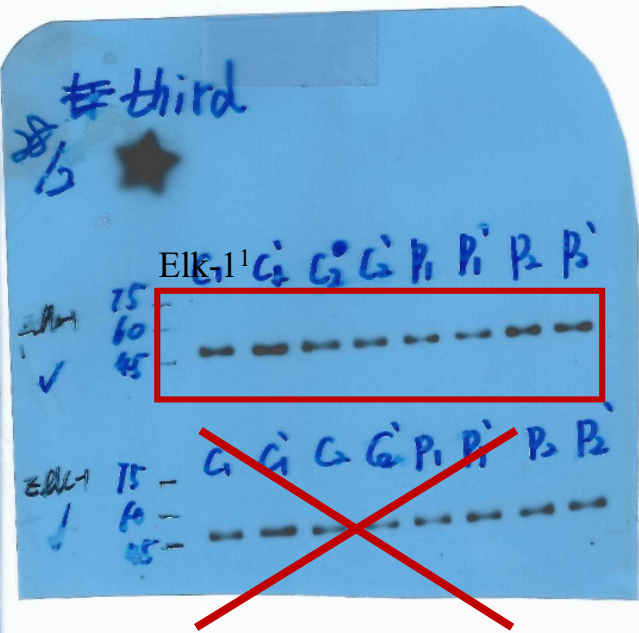

Supplement: S1 Fig — (PDF) [file pone.0284344.s002.pdf]
