# Supplementary material for: A pilot randomized clinical trial of biomedical link with mental health in art therapy intervention programs for alcohol use disorder: Changes in NK cells, addiction biomarkers, electroencephalography, and MMPI-2 profiles
Source: PLoS One. 2023 May 5;18(5):e0284344. doi: 10.1371/journal.pone.0284344 (PMC10162529; doi:10.1371/journal.pone.0284344)
Supplement: S1 Table — (DOCX) [file pone.0284344.s003.docx]

**S1 Table. Restructured clinical scales and Psy-5 scales T-scores were compared by the group.**

| **Classification** | **Control group (*n*=15)**  ***M* (*SD)*** | | | | **Experimental group (*n*=20)**  ***M* (*SD)*** | | | |
| --- | --- | --- | --- | --- | --- | --- | --- | --- |
| **Classification** | **Before** | **After** | ***Z*** | ***p*** | **Before** | **After** | ***Z*** | ***p*** |
| RCd | 56.933 (13.2636) | 52.933 (15.5998) | -1.930 | 0.054 | 57.600 (16.1356) | 49.850 (14.5504) | -2.659** | 0.008 |
| RC1 | 50.800 (6.8159) | 52.600 (6.8013) | -1.197 | 0.231 | 53.600 (11.1232) | 51.350 (12.6002) | -0.982 | 0.326 |
| RC2 | 52.400 (12.2579) | 53.333 (13.9728) | -0.788 | 0.431 | 57.700 (13.4011) | 51.050 (9.6762) | -2.322* | 0.020 |
| RC3 | 48.067 (12.0444) | 47.800 (11.5090) | -0.032 | 0.975 | 51.200 (13.4697) | 45.900 (11.8406) | -2.639** | 0.008 |
| RC4 | 59.467 (10.1268) | 60.133 (9.4330) | -0.281 | 0.779 | 64.950 (13.7782) | 60.450 (13.0484) | -1.677 | 0.093 |
| RC6 | 48.800 (8.2393) | 47.200 (6.3155) | -1.022 | 0.307 | 50.450 (12.4371) | 46.600 (9.1617) | -1.436 | 0.151 |
| RC7 | 50.600 (9.3182) | 48.333 (11.7999) | -0.882 | 0.378 | 55.950 (14.0768) | 48.300 (13.5340) | -2.638 | 0.007 |
| RC8 | 52.933 (13.2151) | 49.533 (7.5957) | -1.006 | 0.314 | 52.100 (15.0224) | 46.600 (8.6292) | -2.138* | 0.033 |
| RC9 | 53.333 (10.6949) | 50.533 (12.9166) | -1.231 | 0.218 | 49.600 (10.9420) | 47.300 (11.5216) | -1.475 | 0.140 |
| AGGR | 50.733 (12.6235) | 47.867 (9.4858) | -0.739 | 0.460 | 46.600 (10.2413) | 45.050 (9.4450) | -0.869 | 0.385 |
| PSYC | 51.600 (10.4458) | 47.867 (6.6318) | -1.542 | 0.123 | 51.450 (16.3529) | 45.900 (10.0729) | -1.539 | 0.124 |
| DISC | 56.667 (12.7036) | 53.333 (1.1254) | -2.036* | 0.042 | 59.300 (11.8193) | 56.650 (14.5901) | -1.308 | 0.191 |
| NEGE | 55.333 (10.5198) | 52.533 (12.8222) | -1.320 | 0.187 | 57.300 (14.0079) | 50.200 (13.6521) | -2.819** | 0.005 |
| INTR | 49.267 (10.0745) | 49.200 (1.5302) | -0.285 | 0.775 | 56.050 (11.6911) | 50.800 (9.4010) | -2.239* | 0.025 |

** *p <*0.01

* *p <*0.05.

RCd (Demoralization), RC1 (Somatic Complaints), RC2 (Low Positive Emotions), RC3 (Cynicism), RC4 (Antisocial Behavior), RC6 (Ideas of Persecution), RC7 (Dysfunctional Negative Emotions), RC8 (Aberrant Experienced), RC9 (Hypomanic Activation), AGGR (Aggressiveness), PSYC (Psychoticism), DISC (Disconstraint), NEGE (Negative Emotionality/Neuroticism), INTR (Introversion/Low Positive Emotionality)
