# Supplementary material for: A pilot randomized clinical trial of biomedical link with mental health in art therapy intervention programs for alcohol use disorder: Changes in NK cells, addiction biomarkers, electroencephalography, and MMPI-2 profiles
Source: PLoS One. 2023 May 5;18(5):e0284344. doi: 10.1371/journal.pone.0284344 (PMC10162529; doi:10.1371/journal.pone.0284344)
Supplement: S2 Table — (DOCX) [file pone.0284344.s004.docx]

**S2 Table. Content scales of T-scores were compared by the group.**

| **Classification** | **Control group (*n*=15)**  ***M* (*SD)*** | | | | **Experimental group (*n*=20)**  ***M* (*SD)*** | | | |
| --- | --- | --- | --- | --- | --- | --- | --- | --- |
| **Classification** | **Before** | **After** | ***Z*** | ***p*** | **Before** | **After** | ***Z*** | ***p*** |
| ANX | 47.600 (7.5951) | 45.533 (11.8011) | -0.346 | 0.730 | 59.850 (15.7656) | 50.100 (14.9874) | -2.900** | 0.004 |
| FRS | 56.067 (4.6975) | 58.200 (7.7201) | -0.700 | 0.484 | 46.700 (11.7119) | 45.700 (10.2449) | -0.655 | 0.513 |
| OBS | 52.467 (8.7003) | 50.533 (8.3910) | -0.350 | 0.726 | 53.950 (13.7744) | 49.300 (14.1351) | -1.993* | 0.046 |
| DEP | 52.200 (11.7850) | 50.933 (9.7429) | -1.610 | 0.107 | 58.150 (14.5178) | 49.050 (12.8533) | -3.121** | 0.002 |
| HEA | 50.200 (7.6270) | 48.133 (6.6748) | -0.909 | 0.363 | 54.200 (10.9765) | 51.450 (10.7971) | -1.431 | 0.152 |
| BIZ | 49.800 (9.8213) | 51.467 (10.9601) | -1.248 | 0.212 | 51.300 (13.6386) | 45.650 (8.0608) | -2.293* | 0.022 |
| ANG | 50.000 (10.7305) | 51.667 (13.2270) | -0.629 | 0.529 | 52.650 (11.1179) | 49.600 (11.4680) | -1.061 | 0.289 |
| CYN | 46.800 (10.3662) | 50.400 (12.9318) | -0.199 | 0.842 | 51.350 (12.9097) | 50.700 (12.0180) | -2.645** | 0.008 |
| ASP | 52.267 (10.0887) | 51.400 (7.5290) | -0.491 | 0.624 | 53.100 (14.4765) | 50.700 (12.0180) | -1.119 | 0.263 |
| TPA | 55.733 (11.8651) | 55.000 (11.3137) | -1.822 | 0.068 | 50.050 (10.8796) | 45.650 (8.0412) | -2.196* | 0.028 |
| LSE | 54.267 (11.4920) | 55.400 (11.2301) | -0.904 | 0.366 | 57.250 (18.2897) | 50.950 (13.3001) | -2.483* | 0.013 |
| SOD | 63.400 (10.2595) | 59.867 (8.1404) | -0.276 | 0.782 | 57.250 (18.2897) | 52.350 (14.7480) | -2.022* | 0.043 |
| FAM | 51.467 (9.0306) | 50.933 (9.4979) | -0.256 | 0.798 | 57.600 (14.3248) | 51.350 (12.2228) | -2.679** | 0.007 |
| WRK | 56.733 (11.0613) | 54.200 (10.7383) | -2.200* | 0.028 | 57.500 (16.8726) | 50.200 (14.0847) | -2.922** | 0.003 |
| TRT | 54.067 (11.9610) | 53.733 (15.3691) | -0.825 | 0.409 | 51.250 (13.0983) | 46.700 (10.8875) | -1.789 | 0.074 |

** *p <*0.01

* *p <*0.05.

ANX (Anxiety), FRS (Fears), OBS (Obsessiveness), DEP (Depression), HEA (Health Concerns), BIZ (Bizarre Mentation), ANG (Anger), CYN (Cynicism), ASP (Antisocial Practices), TPA (Type A), LSE (Low Self-Esteem), SOD (Social Discomfort), FAM (Family Problems), WRK (Work Interference), TRT (Negative Treatment Indicators)
