# Supplementary material for: A pilot randomized clinical trial of biomedical link with mental health in art therapy intervention programs for alcohol use disorder: Changes in NK cells, addiction biomarkers, electroencephalography, and MMPI-2 profiles
Source: PLoS One. 2023 May 5;18(5):e0284344. doi: 10.1371/journal.pone.0284344 (PMC10162529; doi:10.1371/journal.pone.0284344)
Supplement: S3 Table — (DOCX) [file pone.0284344.s005.docx]

**S3 Table. Supplementary scales of T-scores were compared by the group.**

| **Classification** | **Control group (*n*=15)**  ***M* (*SD)*** | | | | **Experimental group (*n*=20)**  ***M* (*SD)*** | | | |
| --- | --- | --- | --- | --- | --- | --- | --- | --- |
| **Classification** | **Before** | **After** | ***Z*** | ***p*** | **Before** | **After** | ***Z*** | ***p*** |
| A | 53.733 (9.5129) | 49.867 (12.9055) | -1.793 | 0.073 | 54.800 (13.7481) | 47.050 (12.7464) | -2.939** | 0.003 |
| R | 49.133 (9.4858) | 49.933 (11.6892) | -0.256 | 0.798 | 50.550 (10.1591) | 53.200 (8.4890) | -1.396 | 0.163 |
| Es | 44.800 (11.5028) | 48.533 (1.9796) | -1.435 | 0.151 | 46.050 (12.7835) | 51.350 (10.1529) | -2.539* | 0.011 |
| Do | 43.867 (10.5008) | 44.600 (12.8885) | -0.223 | 0.824 | 44.150 (9.1839) | 46.950 (10.9231) | -1.535 | 0.125 |
| Re | 46.400 (10.8351) | 48.800 (13.3159) | -0.910 | 0.363 | 46.200 (11.4460) | 49.700 (10.7218) | -1.760 | 0.078 |
| Mt | 55.467 (12.6878) | 52.667 (14.5782) | -0.880 | 0.379 | 58.250 (14.8355) | 48.900 (16.1340) | -2.832** | 0.005 |
| Pk | 55.267 (10.8065) | 52.200 (12.2428) | -1.434 | 0.152 | 58.350 (13.8954) | 50.200 (13.1133) | -3.313** | 0.001 |
| MDS | 52.400 (8.3905) | 52.667 (7.8072) | -0.214 | 0.830 | 56.400 (9.0286) | 49.800 (11.1996) | -2.968** | 0.003 |
| Ho | 49.867 (10.9013) | 46.267 (10.0176) | -1.887 | 0.059 | 50.650 (14.0386) | 46.150 (12.9585) | -1.752 | 0.080 |
| O-H | 52.333 (7.6126) | 50.733 (13.0410) | -0.371 | 0.711 | 48.900 (10.8040) | 51.750 (11.3131) | -1.015 | 0.310 |
| MAC-R | 56.133 (11.3570) | 53.667 (9.5369) | -1.400 | 0.162 | 56.500 (11.9934) | 53.350 (12.9504) | -0.971 | 0.331 |
| AAS | 62.867 (7.7815) | 52.667 (12.0929) | -1.263 | 0.206 | 61.900 (13.2859) | 55.150 (13.0959) | -2.619** | 0.009 |
| APS | 54.200 (14.1229) | 52.667 (12.0929) | -1.298 | 0.194 | 56.350 (12.4320) | 50.000 (11.9781) | -1.913 | 0.056 |
| GM | 48.200 (10.7717) | 49.267 (9.5129) | -0.490 | 0.624 | 47.150 (11.7977) | 50.950 (10.2827) | -1.632 | 0.103 |
| GF | 45.533 (8.3227) | 50.133 (9.5608) | -2.842** | 0.004 | 44.800 (6.9328) | 47.800 (8.9065) | -1.545 | 0.122 |

** *p <*0.01

* *p <*0.05.

A (Anxiety), R (Repression), Es (Ego Strength), Do (Dominance), Re (Social Responsibility), Mt (College Maladjustment), PK (Post-Traumatic Stress Disorder), MDs (Marital Distress), Ho (Hostility), O-H (Overcontrolled-Hostility), MAC-R (MacAndrew Alcoholism-Revised), AAS (Addiction Admission), APS (Addiction Potential), GM (Masculine Gender Role), GF (Feminine Gender Role)
