# Supplementary material for: A pilot randomized clinical trial of biomedical link with mental health in art therapy intervention programs for alcohol use disorder: Changes in NK cells, addiction biomarkers, electroencephalography, and MMPI-2 profiles
Source: PLoS One. 2023 May 5;18(5):e0284344. doi: 10.1371/journal.pone.0284344 (PMC10162529; doi:10.1371/journal.pone.0284344)
Supplement: S1 File — (PDF) [file pone.0284344.s006.pdf]

Detailed summary of the study

<table>
<tr><td rowspan="2">Research Title</td><td>Korean</td><td colspan="4">임상미술치료가 알코올 중독 환자의 MMPI-2 프로파일의 변화와 면역세포 수치 및 스트레스 단백질들의 변화에 미치는 영향</td></tr>
<tr><td>English</td><td colspan="4">Effect of clinical art therapy on changes in MMPI-2 profile, in immune cell levels, and in stress-related proteins of alcoholic patients</td></tr>
<tr><td rowspan="2">Principle Investigator</td><td colspan="2">Name</td><td>Affiliation</td><td>Title</td><td>Field of study</td></tr>
<tr><td colspan="2">Kwang-Hyun Baek</td><td>Biomedical Science</td><td>Professor</td><td>Molecular genetics</td></tr>
</table>

1. Background

Along with a rapid increase in single-person households, the number of patients with alcoholism is also increasing. According to Statistics Korea, the proportion of single-person households in Korea has increased rapidly to 28% in 2015 (Statistics Korea, 2016). Alcohol is an easy way to relieve loneliness, and it is difficult to detect alcohol use disorder early as patients live alone. In many cases, patients are already severely addicted to alcohol notice abnormal symptoms. Alcoholism is caused by multiple factors and has various clinical manifestations. However, there is no specific and single treatment for alcoholism to date. As it is easily recurred compared to other mental illnesses, the patients and their families tend to be easily disappointed and give up treatment (Yong, 1995).

The cost of treatment for alcohol-related disease has increased from 1.7057 trillion won in 2007 to 2.4336 trillion won in 2011. In 2011 Epidemiological Survey of Mental Illness by the Ministry of Health and Welfare, 13.4% of those over the age of 20 (approximately 5.12 million people) are addicted to alcohol. Among them, 'alcohol dependence', which is considered as severe alcohol addiction' is observed in 5.3% of those over the age of 20, which corresponds to 2.03 million people (Ministry of Health and Welfare, 2011).

In the modern days, individuals experience psychological phenomena such as stress, disappointment, alienation, and tension due to various problems of industrial society. Janowsky et al., (2001) has reported that rapid economic and sociocultural changes, various environmental stress, and severe life stress that threaten individuals are closely related to drinking behaviors. Habitual and chronic drinking from increased rate of alcohol consumption

has led to an increased number of patients with alcoholism that causes various problem behaviors (Shin, Jung & Han, 1990).

Kwon (2000) reported that at least one out of five people in Korea will develop alcohol problems during their lifetime (Kwon, 2000). Alcoholism is defined as a state of continuing to excessively drink despite impaired personal health of social and occupational functions and generally includes alcohol abuse and dependence (Hyun, 2002). Alcoholism patients fail to solve realistic anxiety and become habituated to solve problems through alcohol. Thus, they develop an obsessive attitude or thoughts that alcohol can solve every problem. Therefore, alcoholism patients also often develop emotional disorder and have lower tolerance to tension, which makes them impulsive and selfish. In many cases, alcoholism patients have anti-social, dependent, or cyclothymic personality. Such alcoholism is not a simple disease, but a chronic and fatal progressive disease with a high recurrence rate.

Shim, Lee, and Lee (1989) reported that alcoholism is a family disease that impairs the functioning of the family and a social disease that causes social problems such as homicide, suicide, violence, sexual problems, drinking problems, and crime.

In general, alcohol dependence, commonly known as alcohol addiction, is a chronic and progressive psychiatric disease that causes loss of control and social, legal, psychological, and physical problems through continuous alcohol consumption. In particular, alcohol dependence accompanies or causes psychiatric disorders such as anxiety disorders, mood disorders, and dementia, which lead to serious mental disorders including decreased response to treatment and increased recurrence (Nam et al., 2003).

According to the American Medical Association (AMA), alcoholism is a disease characterized by significant physiological, psychological, and social dysfunctions that are directly related to persistent and excessive drinking. Alcoholism was defined as a form of drug dependence that typically causes difficulties in mental function, physical health, and adaptation to the surrounding environment and emphasized as a disease (Lim, 2005). According to Manual on Alcoholism (1973) of the AMA, alcoholism is a disease with a tendency for drinking in which alcohol is consumed until addiction. Alcoholism has a chronic and progressive tendency for relapse and is characterized by physical disabilities, affective disorders, occupational disorders, and social maladaptation due to continuous and excessive drinking (American Medical Association, 1973; Kim, 2002).

Different treatment methods, including psychological treatment of various methods, are suggested for alcoholism rehabilitation. Clinical art therapy is a new type of treatment

combining medicine and art and is considered as a complementary and alternative therapy that evaluates mental and physical health of patients, treats disease, and improves symptoms (Kim, 2006). It brings synergistic effects to medical treatment and improves the quality of life of patients. In addition, clinical art therapy helps to express inner feelings through art media, and the intervention of art facilities verbal expression and relieves potential tension and anxiety. Thus, clinical art therapy helps patients to acquire self-management skills required to solve life problems through art activities. As Winnicott said, 'man only finds himself when he is creating' (Rubin, 2006). Art therapy is the work of recognizing problems and developing one-self by discovering one's own suppression, loss, and distortion through the visual medium of art and integrating these through symbolism and totality of art. In particular, art is an important tool for children who are unfamiliar with language expression or people with disabilities to express one's inner self. Art also has a purifying function, which can help alleviate damaged and unstable emotions (Kim, 2006).

2. Purpose

Alcoholism has a long history and can directly or indirectly affect all members of society regardless of age, gender, education level, socioeconomic status, and residential neighborhood. Patients with alcoholism have a unique negative cognitive system that perceives any factors related to themselves as unrealistic and negative. As such, negative cognitive system in alcoholism patients leads to various problem behaviors. Environmental or genetic stimuli leads to stress responses in almost every tissue in the body, leading to decreased attention and concentration. Additionally, stress is directly correlated to alcohol consumption (North et al., 2011). Most patients start drinking a glass or two of alcohol to forget external stress. Excessive drinking causes alcohol to directly act on tissues involved in stress response, leading to increased secretion of proteins including hormones (Song, 2002). This causes more stress in the body. Such reactions suggest that alcohol dependence may become more severe when patients encounter difficulties in daily life. In treated patients, stress is directly proportional to relapse of drinking. Moreover, continuous consumption of alcohol significantly lowers the amount of all types of white blood cells and reduces the production of antibodies. As a result, patients who consume excessive amounts of alcohol have a lower immune function than normal individuals and are more likely to be infected by external bacterial or viral diseases (Song, 2002).

The purpose of this study to assess changes in stress proteins, which cause alcohol

addiction, as well as in immune cells that may decrease after alcoholism and analyze multiphasic personality inventory to resolve symptoms and various problem behaviors of alcoholism that may be caused by psychological factors.

3. Research institution name and address

KARF St. Mary's Hospital / 86, Ilsan-ro, Ilsandong-gu, Goyang-si, Gyeonggi-do, Republic of Korea, 10450.

4 Name and title of principal investigator, co-investigator, and manager

Principal investigator: Professor Kwang-Hyun Baek / Department of Biomedical Science, CHA University

Co-investigator: Soo-Ji Kang (Ph.D. candidate) / Department of Medicine (Major of clinical art therapy) in General Graduate School, CHA University

5. Study period

12 months from the date of IRB approval (expected end date: March 16, 2018)

6. Participants

The intended participants of this study are as follows.

- Patients who have been admitted to the research institution for less than seven days (from the date of admission)
- Male and female patients over the age of 20
- Patients hospitalized for alcohol use disorder
- Patients who agree to participate in the study
- Patients who can obtain parental consent for the study

Participants will be randomly assigned to experimental and control groups, and the cut-off points of tools used in pre- and post-test will not be used as criteria for selection of participants. This study will be conducted on adult men and women over the age of 20. Additionally, personal factors such as economic ability, educational background, occupation, family relationship, and place of residence will not be considered in selecting the participants.

7. Estimated number of participants and basis of calculation

A total of 120 participants with 60 participants in each of control and experimental groups will be selected. Only a limited number studies on art therapy in alcohol use disorder patients had more than 20 participants, and no study has analyzed immune cells or stress-related proteins for psychological factors. As more male patients are hospitalized in the research institution than female patients, there is a difference in the number of male and female patients. The minimal number of participants required for statistical analysis is 40 to 50. As the number of participants per age group may be adjusted in the future, 70 males and 50 females were calculated to be included in this study.

8. Recruitment of participants

Patients who were hospitalized in the relevant research institution were directly recruited through face-to-face counseling.

9. Participant consent

Consent form with explanations of the study will be prepared, and the purpose, process, method, period, side effects or risks, benefits, and disadvantages to participants, and confidentiality of personal information will be described in the consent form. In addition, the participants will be informed that they can withdraw from the study to convey that they are voluntarily agreeing to participate in the study. The written consent form must be signed by the participant.

10. Methods

Changes in immune cell levels and stress proteins will be assessed after 12 sessions of clinical art therapy. Pre- and post-test will be conducted in both experimental and control groups to collect blood samples and compared the levels of immune cells and stress proteins.

- Recruited participants will be randomly assigned to experimental and control groups. (participants will be randomly assigned as social and occupational characteristics will not be used)
- Pre-test will include MMPI-2, EEG, and evaluation of immune cells and stress protei

ns through blood samples.

- Recruited participants will be randomly assigned to experimental and control groups.
- Pre-test will include MMPI-2, EEG, and evaluation of immune cells and stress protei ns through blood samples.
- The experimental group will undergo 12 sessions of clinical art therapy after pre-test
- Clinical art therapy will be conducted in groups of 5-10 participants, once a week for 60 minutes in a treatment room in the relevant institution.
- During the therapy, the participants will undergo therapy for 40 minutes according tot he goals of each session, and for the remaining 20 minutes, the group will have cha nce to sympathize and communicate with each other by sharing stories about the art.
- After the 12 sessions, the experimental group will undergo same assessments as pre-t est for post-test. The control group will also undergo post-test
- The control group will not receive clinical art therapy and will conduct pre-test after the start of the study and post-test after three months from the start of the study.
- All tests and treatment will be provided within the research institution, and analysis of the tests may be requested to specialized external institutions. All information colle cted from requests of analysis to external institutions will be statistically analyzed an d discarded after storing for three years from the end of the study.
- Changes in immune cells and stress proteins will be analyzed using blood samples co llected in pre- and post-test. Physical responses caused by psychological support from clinical art therapy will be analyzed in alcoholism patients. This study will observe ho w stress proteins changes with psychological support and stability and assess changes in immune cells lowered by various factors

11. Observation items

① Minnesota Multiphasic Personality Inventory 2 (MMPI-2) for adults

MMPI is a representative self-reported personality test developed by Hathaway a nd Mckinley (Hathaway & Mckinley, 1943). Currently, MMPI-2 (Butcher et al., 1989) a nd MMPI-A (Butcher et al., 1992), which are revised versions of original MMPI for ad ults and adolescents, respectively, are used. MMPI-2 consists of a validity scale that re flects attitude during the examination and 10 clinical scales as well as a reconstruct cli

nical scale, personality pathology 5-factor scale, content scale, supplementary scale, and critical items.

MMPI was originally developed to accurately diagnose and evaluate patients undergoing psychiatric treatment in hospitals. Today, MMPI is not only used as a diagnostic evaluation tool, but also quantify and objectively measure specific symptoms experienced by patients, personality traits that affect psychopathology, level of adaptation, and attitude during examination. Furthermore, MMPI is widely used for other purposes such as psychological counseling for normal people, personnel selection, legal advice, mental health-related screening tests, and research.

② EEG (2Channel NeuroHarmony)

EEG test items

- Prediction of self-control index
- Prediction of attention index that indicates brain arousal level and resistance to disease or stress
- Prediction of emotional index that indicates emotional stability and instability
- Prediction of left and right brain balance index
- Prediction of stress index that indicates resistance to disease and energy
- Effects of EEG: allows objective and scientific analysis and evaluation of self-control, attention, emotional, and stress index that may be greatly improved by clinical art therapy.

③ Blood test (changes in immune cell and stress related proteins levels)

- Blood test will include tests for proteins that response to stress and will allow objective and scientific analysis of improved immune function and reduced stress level
- Blood test items: Immune cell Levels (Natural killer cell), stress-related proteins (SAP kinase, etc.)

12. Collection of human materials

• In the experimental group, human materials will be collected once before the start of the first session of clinical art therapy and once after all 12 sessions of clinical art therapy.

• In the control group, human materials will be collected during pre-test after start of the study and post-test at three months after the start of the study.

• Human materials will be collected by the nurses and clinical pathologists of the relevant research institution.

• For collection of human materials, the participants will be restricted from drinking and intense exercises starting 48 hours before collection. Approximately 15ml of blood will be collected and placed in vacutainer treated with ethylene diamine tetra acetic acid (EDTA) immediately after collection. Then, blood will be stored and transported in a container with ice for analysis.

• Human materials with expired retention period determined by the participants will be disposed in accordance with the standards and methods under Article 13 of the 「Waste Management Act. If the study is abnormally terminated due to reasons such as temporary or permanent closure of the relevant institution, material will be transferred in accordance with the procedures stipulated by the law.

• Studies using human materials of the participants will be carried after the approval of the institutional review board of the relevant institution in accordance with the 「Bioethics and Safety Act」. The relevant institution and investigators will take necessary measures to protect personal information of the participants

• All collected information will be disposed within three years from the end of the study.

13. Efficacy evaluation criteria and methods

• Multiphasic personality test and clinical art therapy may help to reduce social problems in alcoholism patients.

• Clinical art therapy may lead to changes in immune cells in patients with alcoholism whose immune function is lower compared to normal healthy individuals.

• Stress is the direct cause of alcohol addiction, and changes in stress proteins can help to develop and manualize clinical art therapy program.

• Medical approach and tests help to observe objective verification effects, and and clin

ical art therapy intervention programs can be a new model to reduce social problems and lower the recurrent rate of alcoholism.

14. Safety evaluation criteria and methods

This study does not involve drug administration and physical damages to the body. Thus, safety evaluation is not applicable.

15. Data analysis and statistical methods

Collected data will be analyzed using SPSS Ver. 12.0 program. Mean and standard deviation will be calculated for all data, and two-way ANOVA with repeated measures will be conducted to assess differences in each measurement variable between the two groups. Turkey HSD (honestly significant difference) method will be used for post-hoc test of significantly different variables.

16. Expected side effects and precautions and measures

- Clinical art therapy does not involve materials that are prohibited for use in hospital (sharp materials and other materials that may threaten lives).
- Excessive immersion and emotional release may lead to physical and mental emergencies such as excitement and hyperventilation. In such cases, clinical art therapy will be stopped immediately, and emergency measures will be provided to help the participant relax.
- Pregnant and lactating women as well as those who are planning to become pregnant during the study period are not allowed to participate.
- Approximately 30mL of blood will be collected from each participant during the study. Blood will be collected in a manner similar to that used in general blood test methods by nurses and clinical pathologists of the relevant research institution. However, improper hemostasis after blood collection may lead to bruises and secondary infections, which may lead to inflammation.
- Risks associated with blood collection are as follows. : Symptoms such as pain, bruises, infection, or inflammation at the site of blood collection and dizziness may be observed. A total of approximately 30mL of blood will be collected over two time points during the study period.

• For any damages from adverse reactions that occur directly through the examinations and clinical art therapy in the course of the study, the participants will be treated by their attending physician, and no additional compensation will be provided.

17. Storage and disposal of human materials

The participants will be restricted from drinking and intense exercises starting 48 hours before tests. Approximately 15ml of blood will be collected and placed in vacutainer treated with ethylene diamine tetra acetic acid (EDTA) immediately after collection. Then, blood will be stored and transported in a container with ice for analysis.

• The participants and their legal representatives can view their consent form and records related to the provision and disposal of human material at any time.
• Human materials with expired retention period determined by the participants will be disposed in accordance with the standards and methods under Article 13 of the 「Waste Management Act」. If the study is abnormally terminated due to reasons such as temporary or permanent closure of the relevant institution, material will be transferred in accordance with the procedures stipulated by the law.
• Studies using human materials of the participants will be carried after the approval of the institutional review board of the relevant institution in accordance with the 「Bioethics and Safety Act」. The relevant institution and investigators will take necessary measures to protect personal information of the participants.

18. Provision of human materials

Human materials collected during the study may be used for research purposes in future studies with the consent of the participants.

The participants cannot claim their rights for product development and patent applications of new drugs or diagnostic tools from studies that used their human materials. Studies that have used human materials provided by the participants will be published in the name of the investigator in conferences and academic journals. Personal information of the participants will be anonymized in numbers for protection.

19. Criteria for discontinuation and dropout

The participants may be withdrawn from the study in following cases.

- The patient voluntarily withdraws from the study
- The patient is judged to have a serious disease and cannot continue in the study
- Those patients who do not originally meet the selection criteria and participate by providing false information, cause serious mental and physical damage through self-harm and harm to others, and seem inadequate for the study at the discretion of the investigator may be restricted from participating in the study.

20. Risks and benefits of participants

During the study, psychological factors that may be observed in recovery of health and alcoholism can be stabilized through intensive clinical art therapy.

The participants may receive better psychological treatment instead and obtain medical knowledge during their hospitalization for the study period. Costs for examinations related to the study (MMPI-2, EEG, blood tests, clinical art therapy) will be covered by the investigators. The participants will undergo psychological tests and receive advices accordingly, and clinical art therapy will improve their understanding of oneself and promote psychological stability. A small souvenir will be provided to the participations after the study as a token of appreciation. This souvenir will not be provided to those who are withdrawn from the study. Improper hemostasis after blood collection may cause bruises and secondary infections, which may lead to inflammation. For any damages from adverse reactions that occur directly through the examinations and clinical art therapy in the course of the study, the participants will be treated by their attending physician, and no additional compensation will be provided.

21. Safety and personal information protection measures for participants

- The investigator will accompany the participants at all times during participation in the study to prepare for emergencies, and any dangerous places or items that are unnecessary will not be used in the study. For any damage related to the study, the participant will be separated and protected for stability. In necessary cases, the participant will be handed over to the guardian or attending physician.
- After emergency first aid, physical and mental treatment will be provided from professionals.

• Participants will not be compensated for any lack of expected therapeutic effects.

• The participants will not be compensated for any damage caused by drug treatment or other treatment and tests that are not directly related to this study.

• For any bodily damage caused by materials in the course of clinical art therapy, treat ment will be provided to the participant at the cost of the investigator.

• All information will be kept confidential to prevent exposure of participant identity. Data will be stored in a locker with locks and will be processes and managed by th e investigators using an encrypted computer. Collected information will be numbered for statistical analysis and discarded after storing for three years from the end of the study. The original artwork of participants from clinical art therapy will be provided to those who wish to receive the work after recording the activity contents.

• Human materials and data collected during the study may be used for research purpo ses in future studies. If the collected materials need to be used for other purposes (in clusion of personal identification information for secondary use), additional consent m ay be obtained.

22. Provision of personal information

Data collected during this study may be used for research purposes in future st udies with the consent of the participants.

23. References

American Medical Association. (1973). Manual on alcoholism. Chicago: the Association.

Butcher, J. N., Dahlstrom, W., Graham, J., Tellegen, A., & Kaemmer, B. (1989). MMPI-2: manual for administration and scoring. Minneapolis: University of Minnesota Press.

Butcher, J.N., Williams, C.L., Graham, J.R., Archer, R.P., Tellegen, A., Ben-Porath, Y.S., & Kaemmer, B. (1992). Minnesota multiphasic personality inventory-adolescent (MMPI-A): Manual for administration, scoring and interpretation. Minneapolis: University of Minnesota Press.

Hathaway, S. R., & McKinley, J. C. (1943). The Minnesota multiphasic personality inventory,

Rev. ed., 2nd printing. Minneapolis: University of Minnesota Press

Hyun, S. J. (2002). Research on the alcoholics' attributional responsibility for the cause and solution of the alcoholic problem. Master's thesis. Seoul; The graduate school of ewha womans university.

Janowsky, D. S., Fawcett, J., Meszaros, K., & Verheul, R. (2001). Core heritable personality characteristics and relapse in alcoholics. *Alcoholism: Clinical and experimental research. 25*(5), 945-985.

Kim, H. S. (2003). A study of al-anon family group for the families of the alcoholic: Centered on the wives of the alcoholic. Master's thesis. Seoul; The graduate school of myonji university.

Kim, S. H. (2006). Clinical Art Therapy. Seoul; Gyechuk munwhasa.

Kwon, S. M. (2000). Depression - a swamp of stagnation and despair. Seoul: Hakjisa.

Lim, Y. S. (2005). A study on the stress and stress coping method of alcoholic inpatients. Master's thesis. Seoul; The graduate school of soongsil university.

Ministry of Health and Welfare. (2011), The epidemiological survey of mental disorders in Korea.

NamKoong. K., An, S. K., Lee, E., Lee, E. H., Lee, C. H., & Lee, B. O. (2003). Neurophysiological measurement of alcohol craving using visual event-related potential. *Journal of korean neuropsychiatric association. 42*(4), 466-475.

North, C. S., Ringwalt, C. L., Downs, D., Derzon, J., & Galvin, D. (2011). Postdisaster course of alcohol use disorders in systematically studied survivors of 10 disasters. *Archives of general psychiatry. 68*(2):173-180.

Rubin, J. A. (1999). Art therapy: An introduction. NC; Taylor & Francis Group.

Shim, J. Y., Lee, Y. S., & Lee, H. L. (1989). A study of characteristics and familial factors of

alcoholics attending alcoholics anonymous. *Korean journal of family practice. 10*(12), 24-37.

Shin, S. C., Jung, H. Y., & Han, S. H. (1990). A clinical study of alcoholic patients admitted to neuropsychiatric ward. *Journal of korean neuropsychiatric association. 29*(6), 1372-1380.

Song, B. J. (2002). Think alcohol. Goyang-si: Korean drinking culture research center.

Statistics Korea. (2016). 2015 Population and housing census sample aggregation results.

Yong, H. R. (1995). Stress situation, perception and coping behavior as a relapse factor of the alchoholics. Master's thesis. Seoul; The graduate school of ewha womans university.

Research Participant Consent Form

Research approval number: 1044308-201612-BR-030-03
Principal investigator: name / Professor Kwang-Hyun Baek, affiliation / Department of Biomedical Science, CHA University, contact / +82-31-881-7134, baek@cha.ac.kr

Co-investigator: name / Soo-Ji Kang (Ph.D. candidate), affiliation / Department of Medicine (Major of clinical art therapy) in General Graduate School, CHA University, contact / +82-31-881-7134, speedrabbit823@gmail.com

※. If you have any questions about this study, experience any risks and discomfort indicated in this consent form, or observe damaged related to the study, please contact the principal investigator above. (Other inquiries and consultations: secretary of Institutional Review Board)

1. This study is conducted for research purposes only.

1) The following describes the content of the clinical trial, your role in the clinical trial, and the process of the clinical trial. Please take you time and consider your participation carefully before you sign the consent form. If you have any questions, you can ask the principal investigator or co-investigator at any time.

2) Before you decide to participate in the study, it is essential that you understand the purpose of the study and what you will be doing in the study. The following content is a detailed explanation of the content of this study and your role if you participate in the study. Before signing the consent form, please read the form and ask any questions you may have to the principal investigator or co-investigator.
The purpose of this study is to examine changes in Minnesota Multi-phasic Personality Inventory-2 (MMPI-2) profile, EEG, immune cell count, and stress-related proteins after clinical art therapy in patients with alcohol addiction.

2. Study method and predicted efficacy and effects

1) Study method

- Common for experimental and control groups

• Both experimental and control group will undergo baseline assessments, including MMPI-2 test, EEG, and evaluation of immune cell and stress protein levels in blood.

• All tests and treatment will be provided within the research institution, and analysis of the tests may be requested to specialized external institutions.

• Collected information will be numbered for statistical analysis and discarded after storing f or three years from the end of the study.

- Experimental group

• The experimental group will undergo 12 sessions of clinical art therapy.

• The therapy will be conducted in groups of 5-10 people once a week for 60 minutes in a treatment room in the research institution.

• During the therapy, the group will undergo therapy for 40 minutes according to the goals of each session, and for the remaining 20 minutes, the group will have chance to sympat hize and communicate with each other by sharing stories about the art.

• After the 12 sessions, the experimental group will undergo same assessments as pre-test (b aseline assessments) for post-test.

- Control group

• The control group will undergo pre-test (baseline assessment). After three months, the cont rol group will undergo post-test.

• The tests will include MMPI-2 test, EEG, and blood tests.

• During the three months of the study, the control group may receive treatment and classes provided at the research institutions. However, clinical art therapy will not be available.

2) Predicted efficacy and effects

- Multiphasic personality test and clinical art therapy may help to reduce social problems in alcoholism patients.
- Clinical art therapy may lead to changes in immune cells in patients with alcoholism whose immune function is lower compared to normal healthy individuals.
- Stress is the direct cause of alcohol addiction, and medical approach and tests to assess changes in stress-related proteins help to observe objective verification effects. The clinical art therapy intervention program can promote changes in psychological factors and can be a new model that can reduce the recurrence rate and social problems of alcohol use disorder.

3. Probability of random assignment to experimental or control groups

1) Participants will be randomly assigned to control or experimental groups in the study.

① A (experimental group) ② B (control group)

- Random assignment

Participants will be randomly assigned to the groups using a computer program (random draw) prior to the start of the study. Random assignment helps to ensure reliable comparison of information collected for the experimental group without the subjectivity of the researcher affecting the assignment of the experimental group. The participants will have 50% change to be assigned to each group.

4. Examinations and procedures in the study

1) Clinical art therapy will be performed once a week for 60 minutes. The therapy will be conducted in groups of 5-10 participants for a total of 12 sessions.
2) The experimental group will undergo the following examinations before the start of the first session of clinical art therapy and after completing all 12 sessions.
The control group will undergo pre-examinations once after the start of the study and post-examinations at three months later.

① MMPI-2

MMPI-2 is a self-reported personality test developed to evaluate personality, emotion, and a daptation level in a multi-dimensional manner. It is widely used for various purposes such as psychological counselling, personnel selection, legal advice, mental health-related screenin g tests, and research.

② EEG (2Channel NeuroHarmony)

EEG test items

- Prediction of self-control index
- Prediction of attention index that indicates brain arousal level and resistance to disease or stress
- Prediction of emotional index that indicates emotional stability and instability
- Prediction of left and right brain balance index
- Prediction of stress index that indicates resistance to disease and energy
- Effects of EEG: allows objective and scientific analysis and evaluation of self-control, atte ntion, emotional, and stress index that may be greatly improved by clinical art therapy.

③ Blood test (changes in immune cell and stress related proteins levels)

- Blood test will include tests for proteins that response to stress and will allow objective and scientific analysis of improved immune function and reduced stress level

3) Approximately 30ml of blood will be collected from the participants for blood tests.

5. Responsibility of study participants

• The experimental group will undergo 60-minute clinical art therapy sessions once a week after the start of the study.

• The total duration of clinical art therapy will be three months (12 sessions).

• The control group will undergo examinations for a total of two times: once after the start of the study and once after three months from start of the study.

(the control group may receive treatment and classes provided at the research institutions; however, clinical art therapy will not be available)

- For collection of human materials, the participants will be restricted from drinking and int ense exercises for 48 hours before the test. Approximately 15ml of blood will be collecte d every time for two times during the study.

6. Unverified experimental aspects of the study.

- Clinical art therapy is commonly used to treat and help psychological difficulties in variou s diseases. In previous studies, no psychological difficulties were caused by clinical art the rapy. However, in those with a risk of physical or mental emergencies such as excitement or hyperventilation due to excessive immersion or emotional release, clinical art therapy will be stopped immediately, and emergency measures will be provided to induce mental and physical sedation.
- After blood collection, pain, burning sensation, and bruises at the site of needle injection may be observed.

7. Risk (side effects) or inconveniences expected to affect the participants (fetus i f the participant is pregnant or infants if the participant is lactating)

- Pregnant and lactating women as well as those who are planning to become pregnant dur ing the study period are not allowed to participate.
- Clinical art therapy is commonly used to treat and help psychological difficulties in variou s diseases, and various studies have demonstrated the effects of clinical art therapy.
- Clinical art therapy does not involve materials that are prohibited for use in hospitals (sha rp materials and other materials that may threaten lives).
- Excessive immersion and emotional release may lead to physical and mental emergencies such as excitement and hyperventilation. In such cases, clinical art therapy will be stopped immediately, and emergency measures will be provided to help the participant relax.
- Approximately 30mL of blood will be collected from each participant during the study. Bl ood will be collected in a manner similar to that used in general blood test methods by n urses and clinical pathologists of the relevant research institution. However, improper hemo stasis after blood collection may lead to bruises and secondary infections, which may lead to inflammation.

• Risks associated with blood collection are as follows: Symptoms such as pain, bruises, inf ection, or inflammation at the site of blood collection and dizziness may be observed. A total of approximately 30mL of blood will be collected over two time points during the study period.
• Approximately 30mL of blood will be collected from each participant during the study.

8. Expected benefits from participation in the study

During the study, psychological factors that may be observed in recovery of health and alcoholism can be stabilized through intensive clinical art therapy.
The participants may receive better psychological treatment instead and obtain medical knowledge during their hospitalization for the study period. Costs for examinations related to the study (MMPI-2, EEG, blood tests, clinical art therapy) will be covered by the investigators. The participants will undergo psychological tests and receive advices accordingly, and clinical art therapy will improve their understanding of oneself and promote psychological stability. A small souvenir will be provided to the participations after the study as a token of appreciation. This souvenir will not be provided to those who are withdrawn from the study.

9. (if the study involves treatment of a disease) Other treatment options for the disease and the potential risks and benefits of treatments

• If you do not participate in the study, other treatment options include psychotherapy (colo r therapy, music therapy, and dance therapy etc.). Clinical art therapy conducted in this st udy is also used as a psychotherapy. In previous studies, no psychological difficulties wer e caused by clinical art therapy. However, in those with a risk of physical or mental eme rgencies such as excitement or hyperventilation due to excessive immersion or emotional r elease, clinical art therapy will be stopped immediately, and emergency measures will be provided to induce mental and physical sedation.

10. Estimated study participation period and approximate total number of partic ipants

1) Number of participants

① This study will be conducted only at KARF St. Mary’s Hospital, and a total of 60-120

patients will be enrolled. The distribution of participants by age may be adjusted later.

2) Participation period/number of visits

① The study is scheduled to start in March 2017 and end in March 2018. The participants will be asked to visit the research institution 14 times (12 sessions of clinical art therapy and pre- and post-examinations).

② Those who agree to participate and satisfy the inclusion criteria will visit the research Institution and meet the investigators once for pre-examination, for three months (once a we ek), and once for post-examination.

③ This clinical trial will approximately take 12 to 14 weeks. The participants will visit the institution approximately 14 times. Each visit will take about 60-90 minutes.

11. Compensation or treatment for study-related damage

• For any damages from adverse reactions that occur directly through the examinations and clinical art therapy in the course of the study, the participants will be treated by their atte nding physician, and no additional compensation will be provided.

12. Monetary compensation for participation, adjustment according to participation, an d additional cost for participants

• Costs for examinations related to the study (MMPI-2, EEG, blood tests, clinical art therap y) will be covered by the investigators. A small souvenir will be provided to the participa tions after the study as a token of appreciation.

13. The participants will be notified for any new information that may affect their will to continue participating in the study.

14. At the discretion of the investigators, the participants may be restricted from the study without their consent, and such participants may refuse to participate in the study by notifying the principal investigator or co-researchers specified in this consent

form.

15. Any new information during the study that may affect the participants will be noti fied to the participants.

▶ I want to be notified ( )

▶ I do not want to be notified ( )

16. The following is for secondary use of human materials and data collected during t he study.

1) Human materials collected during the study may be used for research purposes in future studies

▶ I want my materials to be used in future studies ( )

▶ I want studies to ask for my permission prior to use ( )

▶ I do not want my materials to be used in future studies ( )

2) Data collected during the study may be used for research purposes in future studies

▶ I want my materials to be used in future studies ( )

▶ I want studies to ask for my permission prior to use ( )

▶ I do not want my materials to be used in future studies ( )

3) Inclusion of personal identification information in data for secondary use

▶ Include personal identification information ( )

▶ Do not include personal identification information ( )

17. Participation in the study is voluntary. Participants may withdraw their consent at any time even after deciding to participate. This will not affect medical treatment or l ead to negative consequences for the participants.

18. Confidentiality of identity (access, storage, management, disposal, and protection of participant identity for presentation of study results)

• The investigator will accompany participant at all times during participation in the study t

o prepare for emergencies, and any dangerous places or items that are unnecessary will not be used in the study. For any damage related to the study, the participant will be protected, and in necessary cases, the participant will be handed over to the guardian or attending physician.

- All information will be kept confidential to prevent exposure of participant identity. Data will be stored in a locker with locks and will be processes and managed by the investigators using an encrypted computer. Collected information will be numbered for statistical analysis and discarded after storing for three years from the end of the study. The original artwork of participants from clinical art therapy will be provided to those who wish to receive the work after recording the activity contents.
- Human materials and data collected during the study may be used for research purposes in future studies. If the collected materials need to be used for other purposes (inclusion of personal identification information for secondary use), additional consent may be obtained.
- Test results and personal information obtained during the study will be anonymized and numbered to protect personal information.
- Clinical art therapy is expected to have positive psychological effects; however, participants will not be compensated for lack of expected therapeutic effects.
- The participants will not be compensated for any damage caused by drug treatment or other treatment and tests that are not directly related to this study.
- The participants may be treated for any damage caused by materials during the course of clinical art therapy.
- The participants and their legal representatives can view their consent form and records related to the provision and disposal of human material at any time.
- Human materials with expired retention period determined by the participants will be disposed in accordance with the standards and methods under Article 13 of the 「Waste Management Act」. If the study is abnormally terminated due to reasons such as temporary or permanent closure of the relevant institution, material will be transferred in accordance with the procedures stipulated by the law.
- Studies using human materials of the participants will be carried after the approval of the institutional review board of the relevant institution in accordance with the 「Bioethics an

d Safety Act」. The relevant institution and investigators will take necessary measures to protect personal information of the participants.

• The participants cannot claim their rights for product development and patent applications of new drugs or diagnostic tools from studies that used their human materials. Studies that have used human materials provided by the participants will be published in the name of the investigator in conferences and academic journals. Personal information of the participants will be not disclosed.

You have read and understand this consent form and have received answers for all questions. I hereby confirm with my signature that I voluntarily participate in this st udy

Participant

Contact /

Name / (signature or seal)

Date of consent /

Participant guardian

Contact /

Name / (signature or seal)

Date of consent /

Personnel who has explained the consent form

Name / (signature or seal)

I confirm that I have received sufficient explanations of the consent form from the relavant personnel and voluntarily completed the consent form.

Principle Investigator

Name / (signature or seal)
