# Supplementary material for: A pilot randomized clinical trial of biomedical link with mental health in art therapy intervention programs for alcohol use disorder: Changes in NK cells, addiction biomarkers, electroencephalography, and MMPI-2 profiles
Source: PLoS One. 2023 May 5;18(5):e0284344. doi: 10.1371/journal.pone.0284344 (PMC10162529; doi:10.1371/journal.pone.0284344)
Supplement: S2 File — (PDF) [file pone.0284344.s007.pdf]

연구상세요약서

연구과제명	국문	임상미술치료가 알코올중독 환자의 MMPI-2 프로파일의 변화와 면역세포 수치 및 스트레스 단백질들의 변화에 미치는 영향			
	영문	Effect of Clinical Art Therapy on Changes in MMPI-2 Profile, in Immune Cell Levels, and in Stress-related Proteins of Alcoholic Patients			
연구책임자	성 명		소속	직위	전공분야
	백광현		생명과학대학 의생명과학	교수	분자유전학

1. 연구배경

혼자 사는 1 인가구의 급증과 함께 알코올 중독 환자 또한 늘고 있는데, 통계청에 따르면 국내 전체 가구 중 1 인가구의 비율은 2015 년 28%에 육박할 정도로 빠르게 늘어가고 있다(통계청, 2016). 일단 외로움과 고독감을 달래는 방법으로 술을 선택하기가 쉽고 혼자 살다 보니 알코올 중독 조기발견이 어렵고, 주변에서 이상증세를 알아챌 시점에는 이미 심각한 중독에 빠져있을 경우가 많다. 알코올 중독은 복합적인 요인에 의해 발병하고 임상양상도 다양하다. 그러나 아직까지는 특별하고 단일한 치료방법이 없으며 다른 어떤 정신질환보다도 쉽게 재발하기 때문에 환자와 가족은 쉽게 실망하고 포기하는 경향이 많다(용후란, 1995).

음주 관련 질병 진료비의 경우 200 년 1 조 7057 억원에서 2011 년 2 조 4336 억원으로 급증했고, 2011 년도 보건복지부 정신질환실태 역학조사에 따르면 한국의 20 세 이상의 국민을 기준으로 알코올중독자가 13.4%, 약 512 만 명에 달한다. 이 가운데 중증 알코올중독으로 분류되는 ‘알코올의존’은 5.3%로 무려 203 만 명에 해당한다(보건복지부, 2011).

현대인은 산업사회의 여러 문제들로 인해 스트레스, 허탈감, 소외감, 긴장감과 같은 정신적인 제 현상을 경험하게 된다. Janowsky et al., (2011)은 최근에 급격한 경제, 사회문화의 변천과 다양한 환경적인 스트레스와 개인을 위협하는 심각한

생활스트레스가 음주행동과 밀접하게 관련이 있다고 보고하였으며, 음주율의 증가와 함께 음주가 습관화되고 만성화 되면서 다양한 문제행동을 유발하는 알코올 중독 증세를 나타내는 사람들의 수가 증가되고 있다(신수철 외, 1990).

권석만(2000)은 연구를 통하여 우리나라 사람은 일생 동안 적어도 다섯 명 가운데 한 명은 알코올 문제를 일으킨다고 하였다. 알코올 중독이란 일반적으로 허용되는 양 이상의 음주를 하여 개인의 건강이나 사회적, 직업적 기능에 장애가 있음에도 불구하고 음주를 계속하는 경우를 가리키며 대개는 알코올 남용과 알코올 의존을 포함하여 말한다(현수진, 2002). 알코올중독자들은 현실적인 불안을 해결하지 못하고, 알코올을 통해서 해결하려는 것이 습관화 되어 알코올로 모든 것을 해결 할 수 있다는 강박적인 태도나 사고를 갖게 된다. 그래서 흔히 정서장애를 수반하고 있고, 긴장에 대한 내성이 저하되어 있기 때문에 충동적이며 이기적인 경우가 많고 반사회적 인격이나 의존적 인격, 그리고 순환성 인격을 지닌 경우가 많다. 문제는 이러한 알코올중독은 단순한 질병이 아니고 만성적 진행적 질환이며 재발률이 높은 치명적인 질환이다.

심재용 외(1989)는 알코올중독은 알코올중독 가족 기능에 손상을 주는 가족질환인 동시에 살인, 자살, 폭력, 성문제, 음주문제 및 범죄 등의 사회문제를 일으키는 사회질환으로 보고하고 있다. 일반적으로 알코올중독이라 불리는 알코올 의존은 지속적인 알코올의 사용으로 환자의 조절력을 잃게 하고 사회적, 법적, 심리적, 신체적인 문제를 일으키는 만성적이고 진행성인 정신과적 질환이다. 특히 알코올 의존은 불안장애, 기분장애, 치매 등의 정신과적 질환을 동반하거나 야기하며, 이로 인해 치료 반응성의 감퇴, 재발의 증가 등 심각한 정신질환이 되는 것으로 알려져 있다(남궁기 외, 2003).

미국의학협회(A.M.A)에서는 알코올중독을 지속적이고 과다한 음주와 직접적으로 관련된 상당한 기능장애가 특징적으로 나타나는 질병으로, 그 기능장애란 생리적, 심리적 및 사회적 기능장애를 말하며, 전형적으로 환자의 정신기능, 신체적 건강, 환경에의 적응에 장애를 가져오는 약물의존의 한 형태라고 정의하여 알코올중독을 질병임을 강조하였다(임영실, 2005).

미국의학협회(A.M.A)의 Manual on Alcoholism(1973)에 의하면 알코올 중독이란 음주에 편향된 특징을 가진 질환으로서 음주가 시작되면서 대게 중독사태가 되어야

끝나며 만성적, 진행적으로 재발되는 경향을 가지고 있고 또한 지속적이고 과도한 음주 때문에 신체장애, 정동장애, 직업장애, 사회부적응 등을 수반하는 특징을 가진다고 정의하고 하고 있다(미국의학협회, 1973; 김혜신, 2003).

알코올 중독 재활에는 많은 치료방법들이 제시되고 있고, 심리적인 치료 또한 다양한 방법으로 이루어지고 있다. 임상미술치료는 의술과 미술이 접목된 새로운 형태의 치료법으로 환자의 심신 상태를 평가하거나 질병의 치료 및 증상의 호전을 도모하는 보완대체요법의 한 분야로(김선현, 2006), 의학적인 치료과정에 시너지 효과를 내고 삶의 질을 높이는 역할을 담당한다. 또한 미술매체를 통해 내면의 감정을 표현하게 해 주며 그림의 개입은 언어적 표현을 용이하게 하고, 잠재적 긴장이나 불안을 완화시켜주므로 집단 원들은 미술활동 중 작품을 완성해 가며 더불어 생활문제 해결에 필요한 자기관리능력을 습득할 수 있다. 도널드 위니컷(D. W. Winnicott)은 '사람은 창작하고 있을 때만 자신을 발견한다(Rubin, 2006)'고 하였다. 미술치료는 미술이라는 시각매체를 통해 스스로 본인의 억제, 상실, 왜곡된 부분을 발견하고 미술의 상징성과 전체성을 통해 통합시킴으로써 자신의 문제를 인지하고 발전시켜나가는 작업이다. 특히 미술은 언어 표현이 익숙하지 못한 아이들이나 장애를 가진 사람들에게 자신의 내면을 표현하는데 더욱 편안한 도구로서의 장점을 가지고 있으며, 또한 미술 자체가 정화기능을 가지고 있어 손상되고 불안정한 감정을 완화시키는데 도움을 줄 수 있다(김선현, 2006).

2. 연구목적

알코올 중독현상은 오랜 역사를 가지고 있으며, 연령, 성별, 교육, 사회경제적 지위, 거주 지역에 관계없이 모든 사회구성원들에게 직접적으로나 간접적으로 영향을 미칠 수 있는 현상이다. 알코올중독자들은 부정적인 인지체계로 인해 일상생활에서 자신과 관련 된 것을 비현실적이고 부정적으로 인지하는 독특한 인지 체계를 가지고 있다. 이와 같이 알코올 중독자들은 알코올 중독으로 인한 증상으로 다양한 문제 행동을 야기 시키는데 이는 부정적인 인지체계의 영향인 것으로 알려져 있으며, 환경이나 유전적인 자극을 받으면 거의 모든 조직에서 스트레스 반응이 나타나, 주의력과 집중력이 떨어지게 된다. 또한 스트레스는 알코올 소비량과 정비례의 상관관계를 갖고 있다(North et al., 2011). 대부분이 외부의 스트레스를 잊어버리려는

의도에서 술을 한 두잔씩 시작하며, 장기간 과음이나 폭음을 하게 되면 알코올 그 자체가 스트레스 반응에 관여하는 조직들에 직접 작용하여 호르몬을 포함한 단백질의 분비를 증가시켜 스트레스를 더 심하게 한다(Song, 2002). 이러한 반응은 일상에서 어려운 일에 부딪치면 알코올 의존성이 높아질 수 있다는 것을 이야기 한다. 일단 알코올 중독을 치료했다 하더라도, 또 다른 스트레스를 받으면 음주를 다시 하기 시작하는 음주 재발현상이 정비례로 나타난다. 또한 지속적인 알코올 음주는 모든 종류의 백혈구 양을 현저히 저하시키고, 면역 단백질의 항체의 생성량도 감소시킨다. 그 결과 알코올 과음자들은 정상인에 비하여 훨씬 낮은 면역기능을 보유하여, 외부의 세균성 또는 바이러스성 질환에 걸릴 확률이 훨씬 높다(Song, 2002).

본 연구에서는 알코올중독의 원인이 되고 있는 스트레스 단백질의 변화와 알코올 중독으로 인해 감소할 수 있는 면역력세포의 변화, 다면적인성검사의 척도를 분석함으로써 심리적인 요인의 변화가 가져올 수 있는 알코올 중독 증세 변화와 다양한 문제행동들을 해결하고자 한다.

3. 연구 실시 기관명 및 주소

카프성모병원 / 경기도 고양시 일산동구 백석동 1241

4 연구책임자, 공동연구자, 담당자의 성명과 직명

연구책임자 : 백광현 교수 / 차의과학대학교 생명과학대학 의생명과학과 교수

공동연구자 : 강수지 / 차의과학대학교 일반대학원 의학과 임상미술치료전공 박사과정

5. 연구기간

IRB 승인일로부터 (12) 개월 (종료예정일자 : 2018 년 3 월 16 일)

6. 연구대상자

연구대상자의 범위는 다음과 같다

- 연구기관에 입원중인 환자들로 입원일 기준 7 일이 지나지 않은 환자
- 만 20 세 이상의 성인 남,녀 환자
- 알코올 사용 장애로 입원한 환자
- 본 연구에 동의한 환자

- 본 연구에 보호자의 동의를 받을 수 있는 환자

연구는 실험군과 대조군을 선정하며 무작위 선정으로 배정하며, 사전, 사후검사로 사용되는 척도들의 절단점이 대상자를 선정하는 기준이 되지 않는다. 본 연구는 성인 남녀를 대상으로 하는 연구로 20 세 이상의 성인 남녀로 대상군을 제한한다. 또한 대상자의 경제적인 능력, 학력, 직업, 가족관계, 거주지 등 개인적인 요소는 대상자 선정 시 고려하지 않는다.

7. 예상 연구대상자 수와 산출 근거

연구대상자는 실험군, 대조군 각 60 명으로 총 120 명으로 한다. 미술치료관련 알코올 사용장애 관련 연구에서 대상자 20 명 이상의 연구는 거의 없으며, 심리적 요인에 대한 면역세포나 스트레스관련 단백질을 분석하는 연구가 없다. 연구기관내의 남성 환자의 비율에 비하여 여성 환자가 적게 분포하고 있어 남녀의 인원의 차이가 있다. 본 연구의 통계처리를 위한 최소인원이 40 명-50 명이며, 성별, 연령별분포인원에 대해서는 추후 조정의 여지가 있어 남자 70 명, 여자 50 명의 인원을 산출하였다.

8. 연구대상자 모집

연구대상자는 연구기관에 입원한 환자들을 대상으로 직접모집하며 대면상담으로 진행한다.

9. 연구대상자 동의

연구대상자를 위한 설명서 및 동의서를 작성하며, 연구의 목적, 참여과정, 방법, 기간, 부작용이나 위험성에 대한 내용, 참여자에 대한 이익, 및 불이익, 개인정보의 비밀보장에 대하여 기술한다. 또한 참여 거부나 중도 철회에 대한 결정이 자유롭다는 것을 알려 연구에는 강제성이 없음을 전달한다. 서면동의는 반드시 연구대상자의 자필서명을 받는다.

10. 연구방법

본 연구는 임상미술치료를 12 회기 실시한 후의 면역세포수치 및 스트레스 단백질들의 변화를 보는 연구로 사전, 사후검사로 실험군, 대조군 모두 혈액을 채취하여 면역세포 및 스트레스 단백질들의 수치를 평가 비교한다.

- 연구게시 후 연구에 참가할 대상자를 모집한 후 무작위로 실험군과 대조군을 설정한다. (사회적, 직업적인 특성이 적용되지 않으므로 무작위방법을 실시한다)
- 사전검사로 실험군, 대조군 모두 다면적인성검사(MMPI-2), 뇌파검사, 혈액을 채취하여 면역세포 및 스트레스 단백질들의 수치를 평가한다.
- 연구게시 후 연구에 참가할 대상자를 모집한 후 무작위로 실험군과 대조군을 설정한다.
- 사전검사로 실험군, 대조군 모두 다면적 인성검사(MMPI-2), 뇌파검사, 혈액을 채취하여 면역세포 및 스트레스 단백질들의 수치를 평가한다.
- 사전검사가 진행된 실험군을 대상으로 임상미술치료 12 회기를 실시한다.
- 기관 내에 있는 치료실에서 일주일에 한번씩 60 분 동안 실시하며, 5 명-10 명사이의 집단 미술치료로 진행한다.
- 60 분 중에 40 분은 임상미술치료의 각 회기별 목표에 맞는 프로그램을 진행하며 20 분 동안 작품에 대한 이야기를 나누며 서로 공감하고 소통하는 시간을 갖는다.
- 12 회기의 임상미술치료를 마친 실험군은 사후검사로 사전검사와 같은 검사를 실시한다. 이때 대조군도 검사를 실시한다.
- 대조군은 임상미술치료를 제공받지 않으며 연구시작 후 사전검사 1 회, 3 개월 뒤 사후검사 1 회를 진행한다.
- 모든 검사와 치료는 연구기관 내에서 이루어지며, 검사의 분석은 전문 외부기관에 의뢰할 수 있다. 외부기관에 분석 의뢰 시 수집정보는 모두 번호로 통계처리한 후 연구종료 후 3 년간 보관 후 폐기한다.
- 사전, 사후 검사로 혈액 수집을 통해 면역세포수치 및 스트레스 단백질들의 변화를 분석하고자 하며, 알코올 중독 환자에게 임상미술치료를 실시하며 심리적인 지원을 했을 때 나타나는 신체적인 반응을 연구하고자 한다. 심리적으로 지지감과 안정감을 얻었을 때 스트레스 단백질들의 변화가 어떻게 일어나는지 관찰하며, 여러 가지 요인으로 낮아져 있는 면역세포수치의 변화를 보고자 한다.

11. 관찰 항목

① 미네소타 다면적 인성 검사(Minnesota Multiphasic Personality Inventory: MMPI)
성인용 MMPI-2 실시

MMPI 는 해서웨이와 매킨리 (Hathaway & Mckinley, 1943)가 개발한 대표적인 자기 보고형 성향 검사로, 현재는 원판 MMPI 를 성인 대상으로 개정한 MMPI-2 (Butcher et al., 1989)와 청소년 대상의 MMPI-A (utcher et al.,1992)가 사용되고 있다. MMPI-2 는 수검 태도를 반영하는 타당도 척도와 10 개의 임상 척도, 그 밖에 재구성 임상 척도, 성격 병리 5 요인 척도, 내용 척도, 보충 척도 및 결정적 문항 등으로 구성되어 있다.

MMPI 는 본래 병원 장면에서 정신건강의학과 치료를 받는 환자들을 정확하게 진단, 평가할 목적으로 개발되었다. 오늘날에는 진단 평가 도구로서 뿐만 아니라 환자들이 경험하는 구체적인 증상, 이러한 정신 병리에 영향을 미치는 성격 특성, 적응 수준, 검사에 임하는 태도 등을 수량화해 객관적으로 측정하기 위해 사용되고 있다. 나아가 정상인을 대상으로 한 심리 상담, 인사 선발, 법적 자문, 정신건강 관련 선별 검사, 연구 등 여러 분야에서 광범위하게 활용되고 있다.

②뇌파검사(2Channel NeuroHarmony)
뇌파검사기 검사항목

- 자기조절지수 예측
- 뇌의 각성정도와 질병이나 스트레스에 대한 저항력을 나타내는 주의지수 예측
- 정서적 안정, 불안정상태를 나타내는 정서지수 예측
- 좌우 뇌 균형지수 예측
- 질병에 대한 저항력과 에너지를 나타내는 스트레스지수 예측
- 뇌파검사의 효과: 임상미술치료를 통해 자기조절지수, 주의지수, 정서지수, 스트레스지수가 많이 향상되고 있음을 객관적이며 과학적인 분석과 평가가 가능함.

③ 혈액검사(면역세포수치 및 스트레스 단백질들의 변화)

- 스트레스에 대한 반응 단백질 검사를 의미하며, 임상미술치료를 통해 면역기능의 향상과 스트레스 수치를 낮추는 객관적이며 과학적인 분석과 평가가 가능함.

- 혈액검사 항목 : Immune Cell Levels (Natural killer cell: 자연살해세포), Stress-related Proteins (SAP kinase 등)

12. 인체 유래물 등 수집방법

- 인체 유래물 수집은 실험군은 임상미술치료 1 회기 시작 전 1 번, 12 회의 임상미술치료가 실시된 후 1 번 수집하여 사전, 사후 2 번의 수집이 이루어진다.
- 대조군은 연구시작 후 사전검사 1 회, 3 개월이 지난 후 사후검사 1 회로 2 번 수집이 이루어 진다.
- 인체 유래물 수집은 연구기관의 간호사 및 임상병리사가 진행한다.
- 인체 유래물 수집 시 대상자들은 검사 48 시간 전부터 음주, 격심한 운동을 제한하며 1 회 에 약 15ml 씩 채취하여 EDTA(ethylene diamine tetra acetic acid)가 처리 된 vacutainer 에 담아 채혈직후 얼음으로 채워진 용기에 보관 . 운반하여 분석한다.
- 연구대상자가 결정한 보존기간이 지난 인체 유래물은 「폐기물관리법」 제 13 조에 따른 기 준 및 방법에 따라 폐기되며, 해당 기관의 휴업・폐업 등 해당 연구가 비정상적으로 종료 될 때에는 법에서 정한 절차에 따라 인체 유래물 등을 이관한다.
- 연구대상자의 인체 유래물 등을 이용하는 연구는「생명윤리 및 안전에 관한 법률」에 따라 해당 기관의 기관생명윤리위원회의 승인 후 진행될 것이며 해당 기관 및 연구자는 연구대상자의 개인정보 보호를 위하여 필요한 조치를 취한다.
- 수집정보는 연구 종료 후 3 년 이내에 폐기처분한다.

13. 효과 평가 기준 및 방법

- 알코올 중독환자들의 다면적 인성을 검사하고 임상미술치료를 실시하여 사회적인 문제에 대한 감소를 기대할 수 있다.
- 임상미술치료를 통해 정상인에 비하여 훨씬 낮은 면역기능을 가지고 있는 알코올중독 환 자들의 면역세포의 변화를 기대할 수 있다.
- 알코올 중독의 직접적인 원인이 되는 스트레스의 변화를 알 수 있는 스트레스 단백질들의 변화를 통해 임상미술치료 프로그램 개발과 매뉴얼화를 할 수

있다.

- 의학적 접근과 검사를 통한 객관적인 검증효과를 볼 수 있으며, 심리적인 요인을 통해 사회적인 문제를 감소시키고 재발률을 낮출 수 있는 새로운 모형이 되리라 생각된다.

14. 안전성 평가 기준 및 평가 방법

본 연구는 약물투여 및 투약, 신체적인 손상을 일으키는 연구가 아니므로 안전성의 평가에 해당사항이 없다.

15. 자료 분석과 통계적 방법

본 연구의 자료 분석은 SPSS Ver. 12.0 프로그램을 이용하며, 모든 자료는 평균과 표준편차를 산출하며 각 측정변인 간의 차이검증은 two-way ANOVA with repeated measure 를 적용한다. 유의 한 차이에 대한 사후검증 은 Turkey HSD(honestly significant difference) 방법을 사용한다.

16. 예측 부작용 및 주의사항과 조치

- 임상미술치료를 실시하는 과정에서 병원에서 사용이 금지된 (날카로운 재료, 생명에 위협 이 될 수 있는 재료)는 사용하지 않는다.
- 지나친 몰입 또는 감정 발산을 통해 흥분, 과 호흡 등의 신체적 · 정신적인 위급 상황이 발생할 가능성이 있다. 이 경우 즉시 임상미술치료를 중단하고 연구대상자의 심신 진정을 유도하는 응급조치를 지원한다.
- 임산부, 수유부, 또는 시험 기간 중에 임신계획을 가지고 있는 여성은 이 시험에 참여할 수 없다.
- 연구대상자는 연구기간 동안에 약 30 mL 정도의 혈액을 채취한다. 채혈방법은 병원에서 시행하는 일반적인 혈액검사 방법과 다르지 않고, 연구기관내의 간호사 및 임상병리사가 혈액을 채취한다. 다만 혈액 채취 후 지혈이 적절하게 되지 않은 경우는 멍이 생길 수 있으며, 여기에 2 차적으로 감염이 되면 염증이 발생할 수 있다.
- 채혈과 관련된 위험은 다음과 같다. : 통증, 멍, 채혈 부위의 감염 또는 염증

및 현기증 과 같은 증상이 발생할 수 있다. 연구 기간 동안 2 회, 약 30 mL 의 혈액을 채취한다.

- 연구에 참여하는 과정에서 받게 되는 검사와 임상미술치료를 통해 직접적으로 발생한 이 상 반응 때문에 상해를 입게 되는 경우 주치의를 통해 치료를 제공하며, 별도의 보상은 이루어지지 않는다.

17. 인체 유래물 보관 및 폐기 방법

연구대상자들은 검사 48 시간 전부터 음주, 격심한 운동을 제한하며 1 회에 약 15ml 씩 채취하여 EDTA(ethylene diamine tetra acetic acid)가 처리 된 vacutainer 에 담아 채혈직후 얼음으로 채워진 용기에 보관 . 운반하여 분석한다.

- 연구 대상자는 이 연구 참여와 관련하여 동의서 및 인체 유래물 등의 제공 및 폐기 등에 관한 기록을 본인 또는 법정대리인을 통하여 언제든지 열람할 수 있다.
- 연구대상자가 결정한 보존기간이 지난 인체 유래물은 「폐기물관리법」 제 13 조에 따른 기준 및 방법에 따라 폐기되며, 해당 기관의 휴업·폐업 등 해당 연구가 비정상적으로 종료될 때에는 법에서 정한 절차에 따라 인체 유래물 등을 이관한다.
- 연구대상자의 인체 유래물 등을 이용하는 연구는 「생명윤리 및 안전에 관한 법률」에 따라 해당 기관의 기관생명윤리위원회의 승인 후 진행될 것이며 해당 기관 및 연구자는 귀하의 개인정보 보호를 위하여 필요한 조치를 취한다.

18. 인체 유래물 제공

본 연구를 위해 수집된 인체 유래물은 연구대상자의 동의가 있을 경우 연구목적으로 본 연구 이외에도 사용되도록 제공할 수 있다. 연구대상자의 인체 유래물 등을 이용한 연구결과에 따른 새로운 약품이나 진단도구 등 상품개발 및 특허출원 등에 대해서는 연구대상자가 권리를 주장할 수 없으며, 제공받은 인체 유래물 등을 이용한 연구는 학회와 학술지에 연구자의 이름으로 발표되고 연구대상자의 개인정보는 번호로 식별 처리하여 보안을 유지한다.

19. 중지 및 탈락기준

연구게시 이후 다음과 같은 경우 중지 및 탈락할 수 있다.

- 대상자가 자발적으로 중지하는 경우
- 대상자의 심각한 질환 및 질병으로 더 이상 연구 진행이 어렵다고 판단된 경우
- 연구대상자 기준을 미충족하면서 거짓으로 참여하였거나 심각한 자해ㆍ타해 등으로 연구 대상자의 신체적ㆍ정신적 손상을 야기하는 경우, 그 외 연구자 판단 하에 부적절한 상황 이 우려되는 경우에는 참여가 제한될 수 있다.

20. 연구대상자의 위험과 이익

연구에 참여함으로써 대상자의 심리적인 부분에 맞는 임상미술치료를 집중적으로 진행함으로써 건강회복 및 알코올중독에서 발생될 수 있는 심리적인 요인에 안정을 얻을 수 있다.

일정기간 기관에 입원하는 동안에 심리적으로 더 나은 치료를 받을 수 있고, 의료적인 지식을 얻을 수 있다. 연구과정에서 필요한 검사(미네소타 다면적 인성검사, 뇌파검사, 연구에 필요한 혈액채취, 임상미술치료)의 비용은 연구자가 부담하며, 연구에 참여함으로써 심리검사 및 그에 따른 자문을 얻을 수 있으며, 임상미술치료를 통해 자신에 대한 이해를 높이고 심리적 안정을 도모할 수 있다. 연구 종료 후에 감사의 뜻으로 기념품이 증정되며, 연구 중도탈락 시에는 지급되지 않는다. 혈액 채취 후 지혈이 적절하게 되지 않은 경우는 멍이 생길 수 있으며, 여기에 2 차적으로 감염이 되면 염증이 발생할 수 있다. 연구에 참여하는 과정에서 받게 되는 검사와 임상미술치료를 통한 직접적으로 발생한 이상반응 때문에 상해를 입게 되는 경우 주치의를 통해 치료를 제공받게 되며, 별도의 보상은 이루어지지 않는다.

21. 연구대상자 안전대책 및 개인정보보호대책

- 연구대상자 보호를 위해 연구에 참여하는 모든 시간 동안 연구자가 함께 상주하여 관찰 하며 응급상황에 대비할 것이며, 연구에 불필요한 위험한 장소, 물품 등은 적용하지 않는다. 만약 연구와 관련된 손상이 발생하였을 경우 사고 대상자를 분리 조치하여 보호하면서 안 정을 유도한다. 필요 시 연구대상자의 보호자 또는 치료받고 있는 병원 담당주치의에게 인 계 한다.
- 사고 응급조치 후에는 전문가 연결을 통한 신체적ㆍ정신적 치료를 지원한다.

- 연구대상자에게 기대되는 치료효과가 나타나지 않은 데에 대해서는 보상하지 않는다.
- 본 연구와 직접적인 관련이 없는 약물치료 및 기타 다른 치료 및 검사시에 발생한 손상에 대해서 보상 및 치료를 제공하지 않는다.
- 임상미술치료 실시과정에서 재료에 의해 발생한 신체상의 손상에 대하여 연구대상자에게 치료를 제공하며, 비용은 연구자가 부담한다.
- 모든 정보는 비밀을 보장하여 연구대상자의 신분 노출을 차단하며 자료는 잠금장치가 있는 사물함에 보관하고 암호화되어 있는 컴퓨터를 사용하며 연구자만 연구 자료를 처리하고 관리한다. 수집정보는 모두 번호로 통계처리한 후 연구종료 후 3 년간 보관 후 폐기한 다. 임상미술치료에 따른 작품은 활동내용 기록을 위한 촬영 이후 희망자에 한하여 연구 대상자에게 원본 제공한다.
- 연구과정에서 채취된 검체 및 자료는 연구목적으로 본 연구 이외에도 향후 사용이 될 수 있으며, 연구 이외에 다른 목적으로 쓰게 될 경우(2 차적 사용을 위한 제공 및 취합된 자료 제공 시 개인식별 정보 포함 여부) 추가적인 동의를 받을 수 있다.

22. 개인정보제공

- 본 연구를 위해 수집된 연구자료는 연구대상자의 동의가 있을 경우 연구목적으로 본 연구 이외에도 사용되도록 제공할 수 있다.

23. 참고문헌

권석만(2000). 우울증-침체와 절망의 늪. 서울: 학지사.

김선현(2006). 임상미술치료학. 서울: 계축문화사

김혜신(2002), 알코올중독자 가족을 위한 가족친목모임(Al_Anon)에 관한 연구. 국내석사학위논문. 서울: 명지대학교 대학원

남궁기, 안석균, 이은, 이은하, 이충헌, 이병욱(2003). 시각자극 사건관련 전위를 이용한 알코올 갈망의 신경 생리적 측정. 신경정신의학. 42(4), 466 – 475.

보건복지부(2011). 2011 정신질환실태역할 보고서

신수철, 정한용, 한신호(1990). 신경정신과에 입원한 알코올리즘 환자에 대한 임상적 고찰. 신경정신의학. 29(6), 1372-1380

심재용, 이연수, 양형규, 강정원(1989). 단주친목에 참가하고 있는 알코올중독자의 특성 및 가족요인에 관한 연구, 가정의학회지, 10(12), 24-37.

송병준(2002). 알코올 백과. 고양시: 한국음주문화연구센터

용후란(1995). 알코올중독자의 재발요인으로서의 스트레스 상황, 지각 및 대처방법. 국내석사학위논문. 서울: 이화여자대학교 대학원

임영실(2005). 알코올중독 재원 환자의 스트레스와 대처방식에 관한 연구. 국내석사학위논문. 서울: 숭실대학교 대학원 석사학위논문

통계청(2016). 2015 인구주택 총 조사 표본집계결과.

현수진(2002). 알코올중독자의 음주문제 원인과 해결에 관한 책임귀인 연구, 이화여자대학교 대학원 석사학위논문.

American Medical Association. (1973). Manual on alcoholism. Chicago: the Association.

Butcher, J. N., Dahlstrom, W., Graham, J., Tellegen, A., & Kaemmer, B. (1989). MMPI-2: manual for administration and scoring. Minneapolis: University of Minnesota Press.

Butcher, J.N., Williams, C.L., Graham, J.R., Archer, R.P., Tellegen, A., Ben-Porath, Y.S., & Kaemmer, B. (1992). Minnesota multiphasic personality inventory-adolescent (MMPI-A): Manual for administration, scoring and interpretation. Minneapolis: University of Minnesota Press.

Hathaway, S. R., & McKinley, J. C. (1943). The Minnesota multiphasic personality inventory, Rev. ed., 2nd printing. Minneapolis: University of Minnesota Press

Janowsky, D. S., Fawcett, J., Meszaros, K., & Verheul, R. (2001). Core heritable personality characteristics and relapse in alcoholics. *Alcoholism: Clinical and experimental research. 25*(5), 945-985.

North, C. S., Ringwalt, C. L., Downs, D., Derzon, J., & Galvin, D. (2011). Postdisaster course of alcohol use disorders in systematically studied survivors of 10 disasters.

Archives of general psychiatry. 68(2):173-180.

Rubin, J. A. (1999). Art therapy: An introduction. NC; Taylor & Francis Group.

[차의과학대학교 생명윤리위원회 양식 7]

연구과제 연구대상자 동의서

임상미술치료가 알콜중독 환자의 MMPI-2 프로파일의 변화와 면역세포 수치 및 스트레스관련 단백질들의 변화에 미치는 영향

연구승인번호: 1044308-201612-BR-030-03

책임연구자: 성명 / 백광현 교수, 소속 / 차의과학대학교 생명과학대학 의생명과학과 연락처 / 031-881-7134, baek@cha.ac.kr

공동연구자: 성명 / 강수지 박사과정, 소속 / 차의과학대학교 일반대학원 의학과 임상미술치료전공, 연락처 / 031-881-7134, speedrabbit823@gmail.com

※. 만일 본 연구에 대한 문의사항이 있으시거나 이 동의서에 표기된 위험이나 불편감이 생기는 경우, 또는 연구와 관련된 손상이 발생할 경우, 위의 연구자에게 연락하여 주시기 바랍니다. (기타 문의 및 상담: 생명윤리위원회 간사)

1. 본 연구는 오로지 연구만을 목적으로 수행됩니다.

1) 아래의 내용은 임상시험의 내용과 임상시험에 참여하실 경우, 귀하가 하실 역할과 임상시험 진행과정 등에 대해 설명하고 있습니다. 귀하는 동의서에 서명하시기 전에, 충분한 시간을 가지고 심사숙고 하여 결정하여 주시기 바랍니다. 궁금하신 사항이 있으면 언제든지 시험책임자나 공동연구자에게 질문하셔도 좋습니다.

2) 귀하가 연구에 참가하실 것을 결정하시기 전에 연구가 왜 실시되며 무엇을 어떻게 하게 될지 정확하게 이해하는 것이 중요합니다. 아래의 내용에는 이번 연구의 내용과 이 연구에 참여하실 경우, 귀하가 할 역할 등에 대한 자세한 설명이 들어있습니다. 귀하는 동의서에 서명하기 전에 충분한 시간을 가지고 동의서를 읽으신 후, 궁금하신 사항이 있으시면 언제든지 연구책임자나 공동연구자에게 질문하시기 바랍니다.

이 연구는 알콜중독환자들을 대상으로 임상미술치료 후 미네소타 다면적인성검사(MMPI-2) 프로파일의 변화와 뇌파변화, 면역세포 수치 및 스트레스관련 단백질들의 변화를 보기 위한 연구목적으로 수행되는 연구입니다.

2. 연구방법 및 예측되는 효능 및 효과

1) 연구방법

- 실험군, 대조군 공통
  - 사전검사로 실험군, 대조군 모두 다면적인성검사(MMPI-2), 뇌파검사, 혈액을 체취하여 면역세포 및 스트레스 단백질들의 수치를 평가하게 됩니다.
  - 모든 검사와 치료는 연구기관 내에서 이루어지며, 검사의 분석은 전문 외부기관에 의뢰할 수 있습니다.
  - 수집정보는 모두 번호로 통계처리한 후 연구종료 후 3 년간 보관 후 폐기합니다.
- 실험군
  - 귀하는 임상미술치료 12 회기를 받게 됩니다.
  - 연구 기관 내에 있는 치료실에서 일주일에 한번씩 60 분 동안 실시하며, 5 명-10 명사이의 집단 미술치료로 진행합니다.
  - 50 분 중에 40 분은 임상미술치료의 각 회기별 목표에 맞는 프로그램을 진행하며 20 분 동안 작품에 대한 이야기를 나누며 서로 공감하고 소통하는 시간을 갖게 됩니다.
  - 12 회기의 임상미술치료를 마친 실험군은 사후검사로 사전검사와 같은 검사를 실시합니다.
- 대조군
  - 귀하는 사전검사 1 회, 3 개월 뒤 사후검사 1 회를 받게 됩니다.
  - 검사는 미네소타 다면적인성검사, 뇌파검사, 혈액검사를 받게 됩니다.
  - 연구기간 3 개월 동안 연구기관 내에서 진행하는 치료, 수업을 받으실 수 있습니다. 단 임상미술치료는 제한됩니다.

2) 예측되는 효능 및 효과

- 알코올 중독환자들의 다면적 인성을 검사하고 임상미술치료를 실시하여 사회적인 문제에 대한 감소를 기대할 수 있습니다.

- 임상미술치료를 통해 정상인에 비하여 훨씬 낮은 면역기능을 가지고 있는 알코올중독 환자들의 면역세포의 변화를 기대할 수 있습니다.
- 알코올 중독의 직접적인 원인이 되는 스트레스의 변화를 알 수 있는 스트레스 단백질들의 변화를 통해 의학적 접근과 검사를 통한 객관적인 검증효과를 볼 수 있으며, 심리적인 요인을 통해 사회적인 문제를 감소시키고 재발률을 낮출 수 있는 새로운 모형이 될 수 있습니다.

3. 연구에 사용되는 시험군 또는 대조군에 무작위로 배정될 확률

1)귀하가 임상시험에 참여하게 될 경우, 무작위 배정을 통해 연구에 참여하시게 됩니다.

① A(실험군) ② B(대조군)

- 무작위 배정

귀하가 이 임상시험에 참여할 경우, 귀하가 받으실 치료는 연구 시작 이전에 컴퓨터 프로그램을 통해 무작위(추첨, 임의)로 배정됩니다. 무작위 배정의 목적은 치료군의 배정에 연구진의 주관이 개입되지 않고, 치료군에 대해 수집된 정보를 신뢰성 있게 비교하기 위함입니다. 귀하가 각 군에 배정될 확률은 1/2 입니다.

4. 본 연구에 참여하게 됨으로써 받게 되는 검사 및 절차

1) 일주일에 한번 60 분 동안 임상미술치료를 진행하게 됩니다. 치료는 5-10 명 내외의 집단으로 이루어지며, 총 12 회기의 임상미술치료를 받게 됩니다.

2) 귀하는 대상자로 선정된 후 실험군은 임상미술치료 1 회기 시작하기 전, 임상미술치료 12 회기를 마친 뒤 다음과 같은 검사를 받게 됩니다.

대조군도 연구게시 후 1 회 사전검사, 3 개월 뒤 사후검사를 진행합니다.

① 미네소타 다면적 인성 검사

개인의 성격, 정서, 적응 수준 등을 다차원적으로 평가하기 위해 개발된 자기보고형 성향 검사로 심리 상담, 인사 선발, 법적 자문, 정신건강 관련 선별 검사, 연구 등 여러 분야에서 광범위하게 활용되고 있습니다.

② 뇌파검사(2Channel NeuroHarmony)

뇌파검사를 통하여

- 자기조절지수 예측
- 뇌의 각성정도와 질병이나 스트레스에 대한 저항력을 나타내는 주의지수 예측
- 정서적 안정, 불안정상태를 나타내는 정서지수 예측
- 좌우뇌 균형지수 예측
- 질병에 대한 저항력과 에너지를 나타내는 스트레스지수를 예측 할 수 있습니다.
- 뇌파검사의 효과: 임상미술치료를 통해 자기조절지수, 주의지수, 정서지수, 스트레스지수가 많이 향상되고 있음을 객관적이며 과학적인 분석과 평가가 가능합니다.

③ 혈액검사(면역세포수치 및 스트레스 단백질들의 변화)
- 스트레스에 대한 반응 단백질 검사를 의미하며, 임상미술치료를 통해 면역기능의 향상과 스트레스 수치를 낮추는 객관적이며 과학적인 분석과 평가가 가능합니다.

3)귀하가 이 임상시험에 참여하게 되면, 약 30ml 정도의 혈액을 채취하여 혈액검사를 실시하게 됩니다.

5. 본 연구를 위해 연구대상자가 준수해야 하는 사항

- 실험군은 연구게시 후 일주일에 한번 60 분의 임상미술치료시간에 참여하게 됩니다.
- 실험군의 임상미술치료기간은 12 회기 3 개월입니다.
- 대조군은 연구게시 후 1 번 , 3 개월이 지난 후 1 번, 총 2 번의 사전, 사후검사를 동일하게 진행하게 됩니다. (대조군은 실험기간 동안에 연구기관 내에서 이루어지는 치료들을 받을 수 있습니다. 단, 임상미술치료는 제한되게 됩니다.)
- 인체유래물 수집시 대상자들은 검사 48 시간 전부터 음주, 격심한 운동을 제한하며 1 회에 약 15ml 씩, 연구기간 내에 2 회 채취하게 됩니다.

6. 본 연구의 검증되지 않은 실험적인 측면

- 임상미술치료는 여러 질환에서 심리적인 어려움을 치료하고, 도움을 주는 치료법으로 일반적으로 많이 사용되는 치료법입니다. 선행연구들에 의하면 임상미술치료를 통해 심리적으로 어려움을 겪게 되는 경우의 선행은 없었으나, 지나친 몰입 또는 감정 발산을 통해 흥분, 과 호흡 등의 신체적ㆍ정신적인 위급

상황이 발생할 가능성이 있는 경우 즉시 임상미술치료를 중단하고 연구대상자의 심신 진정을 유도하는 응급조치를 지원합니다.

- 혈액을 채취하기 위하여 바늘을 꽂은 부위의 통증, 화끈거림, 또는 멍이 드는 증상이 보여 질 수 있습니다.

7. 본 실험에 참여함으로써 연구대상자(연구대상자가 임부일 경우 태아, 수유부일 경우 영유아)에게 미칠 것으로 예견되는 위험(부작용)이나 불편사항

- 임산부, 수유부, 또는 시험 기간 중에 임신계획을 가지고 있는 여성은 이 시험에 참여할 수 없습니다.
- 임상미술치료는 여러 질환에서 심리적인 어려움을 치료하고, 도움을 주는 치료법으로 일반적으로 많이 사용되는 치료법이며, 임상미술치료의 효과성에 대한 논문도 다양하게 발표되었습니다.
- 임상미술치료를 실시하는 과정에서 병원에서 사용이 금지된 (날카로운 재료, 생명에 위협이 될 수 있는 재료)는 사용하지 않습니다.
- 임상미술치료 시간에 지나친 몰입 또는 감정 발산을 통해 흥분, 과 호흡 등의 신체적ㆍ정신적인 위급 상황이 발생할 가능성이 있습니다. 이 경우 즉시 임상미술치료를 중단하고 연구대상자의 심신 진정을 유도하는 응급조치를 지원합니다.
- 귀하는 연구기간 동안에 약 30 mL 정도의 혈액을 채취합니다. 채혈방법은 병원에서 시행하는 일반적인 혈액검사 방법과 다르지 않고, 연구기관내의 간호사 및 임상병리사가 혈액을 채취 합니다. 다만 혈액 채취 후 지혈이 적절하게 되지 않은 경우는 멍이 생길 수 있으며, 여기에 2 차적으로 감염이 되면 염증이 발생할 수 있습니다.
- 채혈과 관련된 위험은 다음과 같습니다. : 통증, 멍, 채혈 부위의 감염 또는 염증 및 현기증과 같은 증상이 발생할 수 있습니다. 연구 기간 동안 2 회, 약 30 mL 의 혈액을 채취할 것입니다

8. 이 연구에 참여함으로써 기대되는 이익

- 연구에 참여함으로써 대상자의 심리적인 부분에 맞는 임상미술치료를 집중적으로 진행함으로써 건강회복 및 알코올중독에서 발생될 수 있는 심리적인 요인에 안정을 얻을 수 있습니다.
- 일정기간 기관에 입원하는 동안에 심리적으로 더 나은 치료를 받을 수 있고, 의료적인 지식을 얻을 수 있습니다. 연구과정에서 필요한 검사(미네소타 다면적인성검사, 뇌파검사, 연구에 필요한 혈액채취, 임상미술치료)의 비용은 연구자가 부담하며, 귀하는 연구에 참여함으로써 심리검사 및 그에 따른 자문을 얻을 수 있으며, 임상미술치료를 통해 자신에 대한 이해를 높이고 심리적 안정을 도모할 수 있습니다. 연구 종료 후에 감사의 뜻으로 기념품이 증정됩니다.(연구 중도탈락 시에는 지급되지 않습니다.)

9. (실험이 질병의 치료와 관련이 있을 경우) 이 질환으로 선택할 수 있는 다른 치료방법 및 이러한 치료의 잠재적 위험과 이익

- 귀하가 연구에 참여하지 않을 경우 귀하가 선택할 수 있는 다른 치료방법으로는 심리치료(컬러테라피, 음악치료, 무용치료 등)가 있습니다. 연구에서 행해지는 임상미술치료도 심리치료방법의 일환으로 적용되고 있습니다. 선행연구들에 의하면 임상미술치료를 통해 심리적으로 어려움을 겪게 되는 경우의 선행은 없었으나, 지나친 몰입 또는 감정 발산을 통해 흥분, 과 호흡 등의 신체적ㆍ정신적인 위급 상황이 발생할 가능성이 있는 경우 즉시 임상미술치료를 중단하고 연구대상자의 심신 진정을 유도하는 응급조치를 지원합니다.

10. 예상 참여기간 및 본 연구에 참여하는 대략의 전체 연구대상자수

1) 연구에 참여하는 피험자 수

① 이 연구는 카프성모병원에서만 시행되며, 총 60-120 명의 환자가 등록될 것이며, 연령별 인원의 분포는 추후 조정될 여지가 있습니다.

2) 참여기간/방문횟수

① 이 연구는 2017 년 3 월에 시작해서 2018 년 3 월까지로 예정되어 있습니다. 귀하는 연구기간 동안 대략 14 회(임상미술치료 12 회, 사전사후검사 2 회)를 방문하셔야 합니다.

②귀하가 연구 참여에 동의하시고, 이 연구 참여에 적합할 경우, 사전검사를 위한 방문 1 회, 이후 연구 기간 3 개월(일주일에 1 번), 사후검사를 위한 방문 1 회 동안 연구자를 만나게 됩니다.
③임상시험의 연구기간은 대략 12-14 주의 기간이 소요됩니다. 연구기간 동안 대략 14 회 병원을 방문하셔야 됩니다. 1 회 병원 방문 시, 약 60-90 분이 소요 됩니다.

11. 연구와 관련된 손상이 발생하였을 경우 연구대상자에게 주어질 보상이나 치료방법

• 연구에 참여하는 과정에서 받게 되는 검사와 임상미술치료를 통한 직접적으로 발생한 이상반응 때문에 상해를 입게 되는 경우 주치의를 통해 치료를 제공받게 되며, 별도의 보상은 이루어지지 않습니다.

12. 연구에 참여함으로써 받게 되는 금전적 보상 유무 및 참여정도에 따른 조정 정도 및 연구에 참여함으로써 연구대상자에게 추가적으로 발생이 예상되는 비용

• 연구에 관련한 검사비용(다면적인성검사, 뇌파검사, 연구를 위한 혈액검사, 임상미술치료)은 연구자가 부담하며, 연구종료 후 감사의 뜻으로 소정의 기념품이 지급됩니다.

13. 연구의 지속 참여 의지에 영향을 줄 수 있는 새로운 정보가 얻어지면 적시에 연구대상자 본인 또는 대리인에게 알려드릴 것입니다.

14. 연구진이 필요하다고 판단할 경우, 본인의 동의 없이도 본 연구 참여에서 제한될 수 있습니다. 또한 이 경우 연구책임자 또는 동의서상에 명시된 연구자에게 통보함으로써 본 연구에서 불참할 수 있습니다.

15. 본 연구진행 중 본인에게 영향을 줄 수도 있는 새로운 정보를 연구자가 획득 시 그 내용을 통보 받을 수 있습니다.

▶ 통보를 원합니다. ( )

▶ 통보를 원치 않습니다. ( )

16. 연구과정에서 채취된 인체유래물 및 자료의 2 차적 사용을 위한 제공에 대한 것입니다.

1) 연구과정에서 채취된 인체유래물은 연구목적으로 본 연구 이외에도 향후 사용될 수 있습니다

▶ 사용을 원합니다. ( )

▶ 사안 발생 시 본인에게 사용허락을 받기 원합니다. ( )

▶ 사용을 원치 않습니다. ( )

2) 연구과정에서 취합된 자료는 연구목적으로 본 연구 이외에도 향후 사용될 수 있습니다

▶ 사용을 원합니다. ( )

▶ 사안 발생 시 본인에게 사용허락을 받기 원합니다. ( )

▶ 사용을 원치 않습니다. ( )

3) 2 차적 사용을 위한 제공 시 개인식별정보 포함 여부

▶ 개인식별정보 포함 ( )

▶ 개인식별정보 불포함 ( )

17. 연구에 대한 귀하의 참여는 자발적입니다. 귀하가 참여하기로 결정한 경우에도, 언제든지 귀하는 자유롭게 연구를 중단할 수 있습니다. 이것은 귀하의 향후 의학적 치료에 어떤 영향을 주지 않고, 아무런 불이익도 받지 않을 것입니다.

18. 신분의 비밀보장 (자료의 열람 권한 설정 및 보관, 관리, 폐기, 연구 결과 발표 시 연구대상자의 신원 보호)

- 귀하는 연구에 참여하는 모든 시간동안 연구자가 함께 상주하여 관찰하며 응급상황에 대비할 것이며, 연구에 불필요한 위험한 장소, 물품 등은 적용하지 않습니다. 만약 연구와 관련된 손상이 발생하였을 경우 사고 대상자를 분리 조치하여 보호하면서 안정을 유도하고, 필요시 연구대상자의 보호자 또는 치료받고 있는 병원 담당주치의에게 인계합니다.
- 모든 정보는 비밀을 보장하여 연구대상자의 신분 노출을 차단하며 자료는 잠금장치가 있는 사물함에 보관하고 암호화되어 있는 컴퓨터를 사용하며 연구자만 연구 자료를 처리하고 관리하게 됩니다. 수집정보는 모두 번호로

통계처리한 후 연구종료 후 3 년간 보관 후 폐기합니다. 임상미술치료에 따른 작품은 활동내용 기록을 위한 촬영 이후 희망자에 한하여 연구 대상자에게 원본 제공합니다.

- 연구과정에서 채취된 검체 및 자료는 연구목적으로 본 연구 이외에도 향후 사용이 될 수 있으며, 연구 이외에 다른 목적으로 쓰게 될 경우(2 차적 사용을 위한 제공 및 취합된 자료 제공 시 개인인식별정보 포함 여부) 추가적인 동의를 받을 수 있습니다.
- 연구과정에서 얻어지는 검사결과 및 개인정보는 익명 및 번호표기하여 개인의 신상정보가 노출되지 않습니다.
- 귀하에게 임상미술치료를 통하여 심리적인 긍정적인 효과를 기대하지만, 기대되는 치료효과가 나타나지 않은 것에 대해서는 보상하지 않습니다.
- 본 연구와 직접적인 관련이 없는 약물치료 및 기타 다른 치료 및 검사 시에 발생한 손상에 대해서 보상하지 않습니다.
- 귀하는 임상미술치료 실시과정에서 재료에 의해 발생한 신체상의 손상에 대하여 치료를 제공받을 수 있습니다.
- 귀하는 이 연구 참여와 관련하여 귀하의 동의서 및 귀하의 인체유래물등의 제공 및 폐기 등에 관한 기록을 본인 또는 법정대리인을 통하여 언제든지 열람할 수 있습니다.
- 귀하가 결정한 보존기간이 지난 인체유래물은「폐기물관리법」제 13 조에 따른 기준 및 방법에 따라 폐기되며, 해당 기관의 휴업·폐업 등 해당 연구가 비정상적으로 종료될 때에는 법에서 정한 절차에 따라 인체유래물 등을 이관할 것입니다.
- 귀하의 인체유래물 등을 이용하는 연구는「생명윤리 및 안전에 관한 법률」에 따라 해당 기관의 기관생명윤리위원회의 승인 후 진행될 것이며 해당 기관 및 연구자는 귀하의 개인정보 보호를 위하여 필요한 조치를 취할 것입니다.
- 귀하의 인체유래물 등을 이용한 연구결과에 따른 새로운 약품이나 진단도구 등 상품개발 및 특허출원 등에 대해서는 귀하의 권리를 주장할 수 없으며, 귀하가 제공한 인체유래물 등을 이용한 연구는 학회와 학술지에 연구자의 이름으로 발표되고 귀하의 개인정보는 드러나지 않을 것입니다.

본인은 이 동의서를 읽고 이해하였으며 모든 질문에 대한 답변을 들었습니다. 이에 본인은 자발적으로 본 연구에 참여함을 서명으로 확인합니다.

연구대상자

연락처 /

성명 / (서명 또는 인)

동의일자 /

대상자 보호자

연락처 /

성명 / (서명 또는 인)

동의일자 /

본인은 상기 연구대상자가 동의서 설명자로부터 동의서에 대한 충분한 설명을 듣고 아무런 강압 없이 전적으로 자발적으로 동의서를 작성하였음을 확인합니다.

연구책임자

성명 / (서명 또는 인)
